# Supplementary material for: Disentangling molecular alterations from water-content changes in the aging human brain using quantitative MRI
Source: Nat Commun. 2019 Jul 30;10:3403. doi: 10.1038/s41467-019-11319-1 (PMC6667463; doi:10.1038/s41467-019-11319-1)
Supplement: Supplementary file 1 — Supplementary Information [file 41467_2019_11319_MOESM1_ESM.pdf]

Supplementary Materials for

"Disentangling molecular alterations from water-content changes in the aging human brain using quantitative MRI"

Shir Filo[†], Oshrat Shtangel[†], Noga Salamon, Adi Kol, Batsheva Weisinger, Sagiv Shifman, and Aviv A Mezer

Correspondence to: aviv.mezer@elsc.huji.ac.il

Table of Content

Supplementary Table 1 3
Supplementary Figure 1 4
Supplementary Note 1 6
Supplementary Note 2 7
Supplementary Note 3 7
Supplementary Figure 2. 8
Supplementary Figure 3. 9
Supplementary Figure 4. 10
Supplementary Figure 5 11
Supplementary Figure 6 12
Supplementary Figure 13
Supplementary Figure 8. 14
Supplementary Figure 9. 15
Supplementary Note 4 16
Supplementary Figure 10. 17
Supplementary Figure 11. 18
Supplementary Figure 12 19
Supplementary Table 2 20
Supplementary Figure 13 21
Supplementary Figure 14. 22
Supplementary Figure 15 23
Supplementary Figure 16 24
Supplementary Figure 17 25
Supplementary Figure 18. 26
Supplementary Note 5 27
Supplementary Figure 19 28
Supplementary Note 6. 29
Supplementary Figure 20. 30
Supplementary Figure 21. 31
Supplementary Figure 22. 32
Supplementary Figure 23. 33
Supplementary Figure 24. 34
Supplementary Figure 25. 35
Supplementary Figure 26. 36
Supplementary Figure 27 37
Supplementary Figure 28. 38
Supplementary Figure 29. 39
Supplementary Figure 30. 40
References 41

Supplementary Table 1 – Glossary of terms

MDM	Multidimensional Dependency on MTV
MTV	Lipid and Macromolecular Tissue Volume
MTsat	Magnetization Transfer saturation
PC	Principal component
PS	Phosphatidylserine
PtdCho	Phosphatidylcholine
PtdCho-Chol	Phosphatidylcholine-cholesterol
PI-PtdCho	Phosphatidylinositol-phosphatidylcholine
Spg	Sphingomyelin
PE	Phosphatidylethanolamine

Supplementary Figure 1 – MDM linearity in pure lipids, lipid mixtures, proteins and sugars.

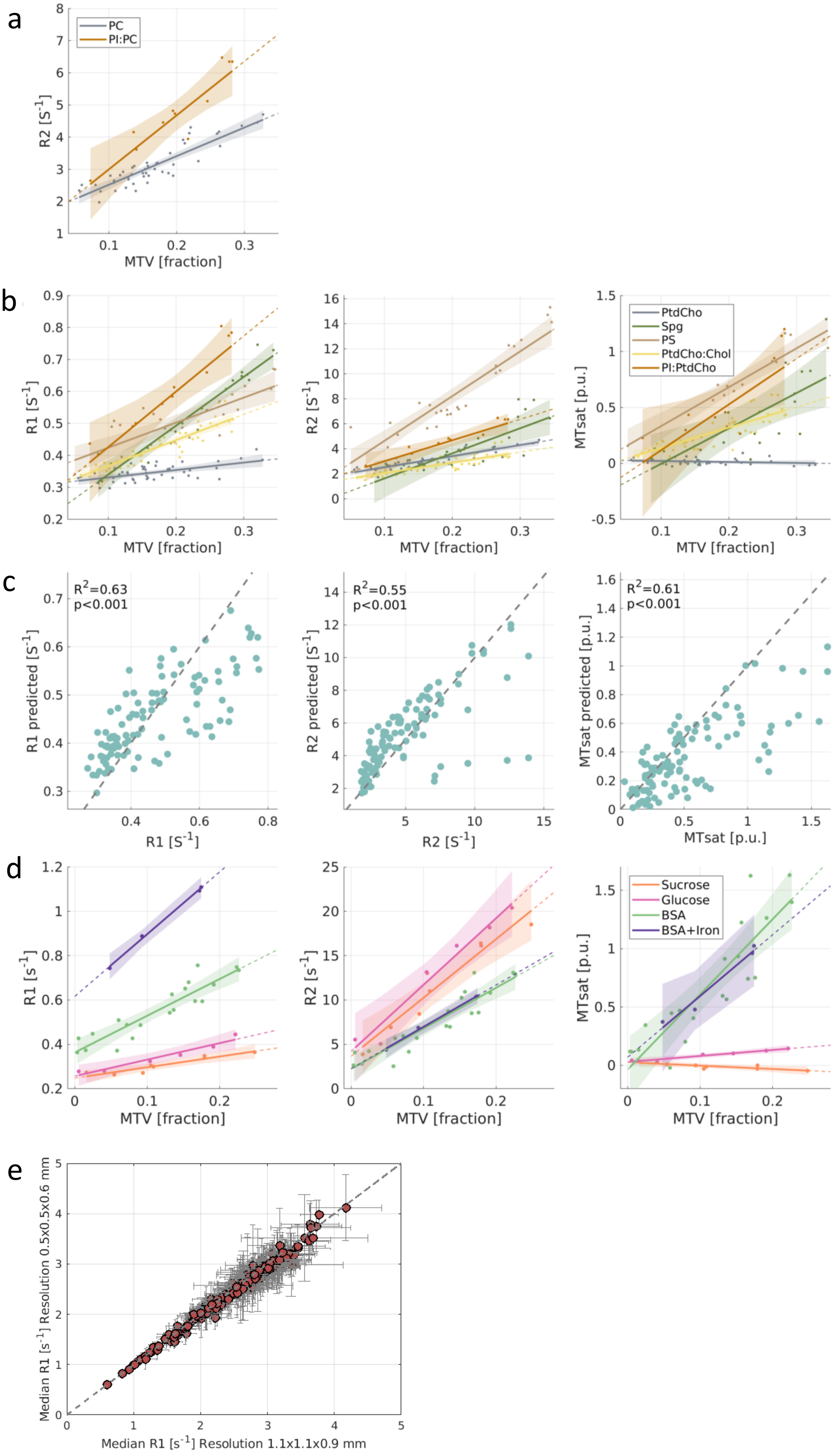

Supplementary Figure 1: MDM linearity in pure lipids, lipid mixtures, proteins and sugars.
a) *The dependency of R2 on MTV for two lipids; phosphatidylcholine (PtdCho) and Phosphatidylinositol-phosphatidylcholine (PI-PtdCho). Colored dots represent the median of different lipid samples with varying water concentrations. The linear relationships between R2 and MTV were fitted according to these data points and are marked by lines. Shaded areas represent the 95% confidence bounds for the fitted slopes.*
b) *The dependency of R1, R2 and MTsat on MTV for 5 lipids (figure properties are as in a).*
c) *Prediction for the R1, R2 and MTsat of 9 lipid mixtures (y-axes) vs. their true values (x-axes). Prediction was evaluated from a linear sum of the MTV dependencies of the pure lipids. For each type of mixture, we scanned different samples with varying water concentrations. All these samples are represented by colored data points. Dashed line is the identity line. Adjusted R^2 values presented.*
d) *The dependency of R1, R2 and MTsat on MTV for non-lipid compounds; two sugars (Sucrose and Glucose), a protein (BSA- Bovine Serum Albumin), and a mixture of protein with iron (BSA+Iron).*
e) *Comparison of median R1 values of different lipid samples acquired in two different resolutions. MRI data used for the computation of R1 and MTV was acquired in two different resolutions (1.1 mm x 1.1 mm x 0.9 and 0.5 mm x 0.5 mm x 0.6). The median R1 values of different lipid samples in these two resolutions shows great agreement.*

Supplementary Note 1- Modeling lipid mixtures through the MDM approach

In order to predict the qMRI parameters of a lipid mixture from MDM measurements (Figure 1d) we used the following model (shown here for MTsat. The predictions for R1 and R2 was done similarly):

$$(1)\ \ MTsat = \sum_{i=1}^{L} f_i(MTsat'_i * MTV + b_i)$$

Where L is the number of lipids in the mixture and f_i is the fraction of the i'th lipid from the total lipid volume. $MTsat'_i$ and b_i are the MDM measurements of the pure lipids. These measures were estimated from the samples prepared exclusively with each i'th lipid and were extracted from the linear fit of these samples:

$$(2)\ \ MTsat_i = MTsat'_i * MTV + b_i$$

Importantly, this model implies that qMRI parameters of a lipid mixture can be computed as a linear sum of qMRI parameters of the pure lipids.

Supplementary Equation 2 holds also for the $qMRI$ parameters of lipid mixtures (Supplementary Figure 2a). Substituting this in Supplementary Equation 1:

$$MTsat'_{mix} * MTV + B_{mix} = \sum_{i=1}^{L} f_i(MTsat'_i * MTV + b_i)$$
$$= \left(\sum_{i=1}^{L} f_i * MTsat'_i\right) * MTV + \sum_{i=1}^{L} f_i * b_i$$

Where $MTsat'_{mix}$ and Bss_{mix} were extracted from the linear fit of the mixture. As this linear equation holds for large range of MTV values and due to the uniqueness of the interpolating polynomial:

$$(3)\ \ MTsat'_{mix} = \sum_{i=1}^{L} f_i * MTsat'_i$$

Therefore, MDM msseasurements of a lipid mixture can be computed as the weighted sum of the MDM measurements of the pure lipids.

Note that since Supplementary Equation 3 holds for the MTV derivative of several qMRI parameters, we can rewrite it in a matrix form:

$$(4)\ \ [\mathrm{M}_{mix}] = [M_{pure}] * [\mathrm{F}]$$

Where M_{mix} is a vector of MDM measurements of a lipid mixture, F is a vector of the lipid fractions of the mixture, and M_{pure} is a matrix of the MDM measurements of the pure lipids. For example, a mixture of PS and Spg with ratios of 2:1 respectively can be represented by the following equation:

$$\begin{bmatrix} R1'_{mix} \\ MTsat'_{mix} \\ R2'_{\mathrm{mix}} \end{bmatrix} = \begin{bmatrix} R1'_{PS} & R1'_{Spg} & R1'_{PtdChol} \\ \mathrm{MTsat}'_{PS} & \mathrm{MTsat}'_{Spg} & \mathrm{MTsat}'_{PtdChol} \\ R2'_{PS} & R2'_{Spg} & R2'_{PtdChol} \end{bmatrix} * \begin{bmatrix} 2 \\ 1 \\ 0 \end{bmatrix}$$

Where $R1'$, $MTsat'$, and $R2'$ are the derivatives of these qMRI parameters with respect to MTV (MDM).

We extended this system to represent several mixtures simultaneously by adding columns describing these mixtures to $[\mathrm{M}_{mix}]$ and $[\mathrm{F}]$. In this case, F is a 3X12 matrix of the lipid composition of each mixture (9 two-lipid samples, and 3 single-lipid samples). The columns of F are different mixtures, and the rows are the volume-based fractions of different lipids. M_{mix} is a 3X12 matrix of MDM measurements. The rows of M_{mix} are the MTV derivatives of R1, R2

and MTsat, and the columns are different mixtures. M_{pure} is a 3X3 matrix with the MDM measurements of pure lipids. The rows of M_{pure} are different MDM measurements, and the columns are different lipids.

Supplementary Note 2- MDM-based Prediction for the lipid composition of a mixture

Supplementary Equation 4 can be transformed to allow prediction of the lipid fractions of a mixture from MDM measurements:

$$(5)\quad [\mathrm{F}] = [M_{pure}]^{-1} * [\mathrm{M}_{mix}]$$

Figure 1e shows that this model provides a good approximation for the lipid composition of a mixture. Importantly, we show that this model does not require prior knowledge about the specific lipid constituents. The mixtures we prepared were composed of two lipids, while the M_{pure} matrix used for prediction incorporated the MDM measurements of three pure lipids.

Supplementary Note 3- MDM-based Prediction for the molecular composition of the human brain

The human brain is far more complex than a lipid mixture. As it is more difficult to a priori estimate the matrix M_{pure} for brain tissue, we show that it is possible to evaluate it through cross-validation.

First, we validated this approach on lipid samples. In each iteration, we fitted M_{pure} using $[\mathrm{F}]$ and $[\mathrm{M}_{mix}]$ of 11 mixtures. We than used the M_{pure} estimate to predict the fractions of the left-out mixture. Through this process, we were able to achieve good estimations for the composition of the lipid mixtures (Supplementary Figure 2b). Moreover, as expected from the theory, the elements of the M_{pure} matrix we fitted are similar to the MDM measurements of pure lipids (Supplementary Figure 2c).

Next, we used the MDM approach to predict the molecular composition of the human brain (Figure 3c). For this, we used Supplementary Equation 5 and the same cross-validation process; prediction for each brain area was computed by removing it from the system and solving for the other brain areas.

The calculation involved 7 human brain molecular features (%PE, %PS, %PtdCho %PI, %Spg, phospholipids/proteins, phospholipids/cholesterol), and 4 MDM measurements (dR1/dMTV, dMTsat/dMTV, dR2dMTV, dMD/dMTV). PCA was used to reduce the dimensionality of the system and avoid over-fitting. We identified the three molecular features with the largest loadings on the first PC of molecular variability. The fractions of the lipids PE and PtdCho had the largest loadings, and we used the ratio between them as it was found to better characterize individual brain regions [1]. The two other features with large loadings were the fraction of the lipid Spg and the phospholipids/proteins ratio. We predicted these 3 human brain molecular features using the MTV derivatives that account for most of the MDM variability. The two measurements with the largest loadings on the first PC of MDM were dR1/dMTV and dMTsat/dMTV.

Therefore, in the case of the human brain, F is a 3X7 matrix of molecular composition estimated from the literature [1]. The columns of F are 7 different brain areas. The rows of F are the 3 molecular features with largest loadings. M_{mix} is a 2X7 matrix of the MDM measurements of 7 different brain regions. This data was calculated from the MRI measurements averaged over the young subjects. The rows of M_{mix} are two MDM measurements with large loadings on the first PC of MDM variability.

Supplementary Figure 2 – MDM-based prediction for lipid samples with cross validation.

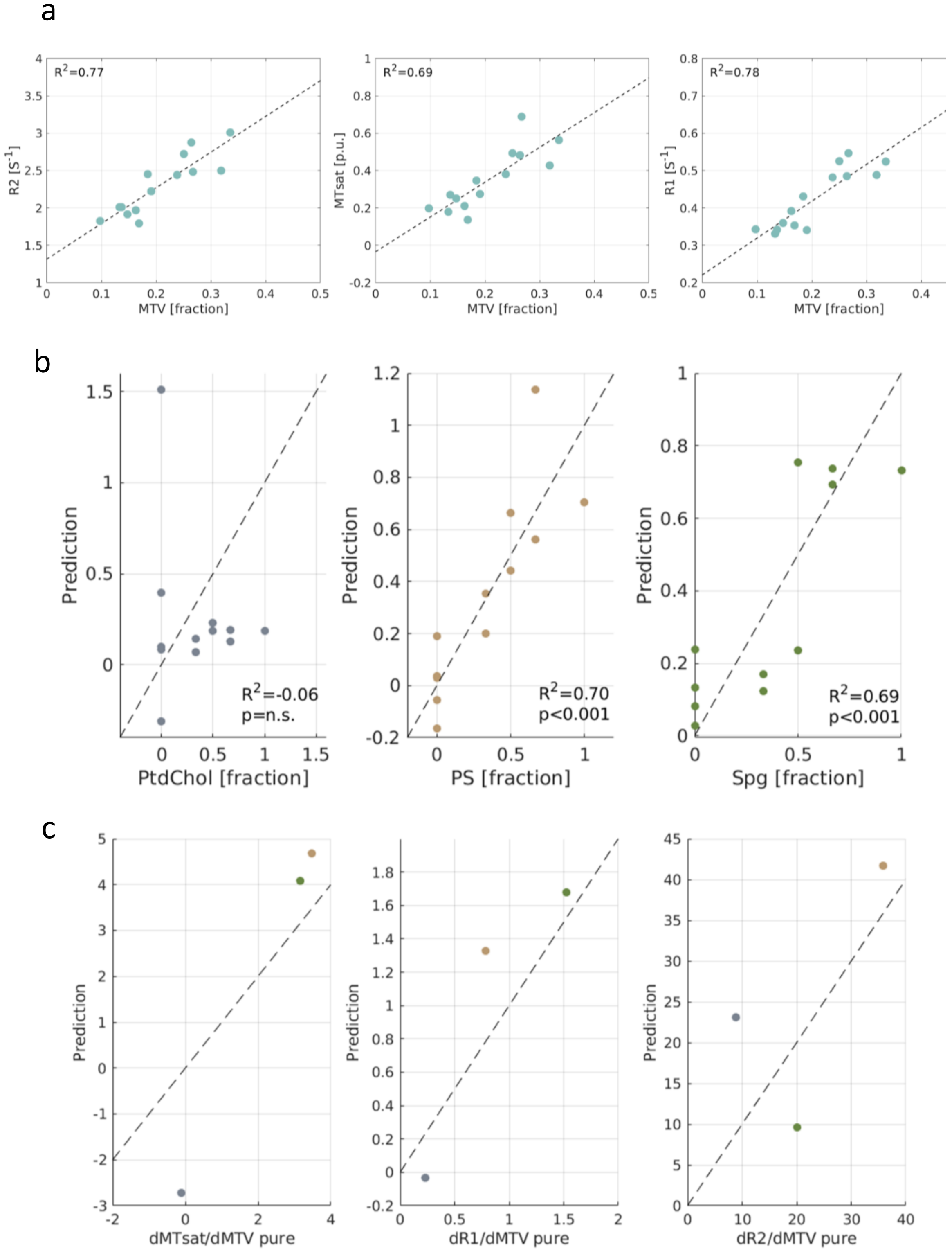

Supplementary Figure 2: MDM-based prediction of the lipid composition in mixtures using cross validation.
a) *The linear dependeny of qMRI parameters on MTV in a lipid mixture comprising Ptd-Cho:Spg with ratio 1:2. Data points represent the median of different lipid samples with varying water concentrations. The linear relationships between MTV and R2 (right), MTsat (middle) and R1(left) were fitted according to these data points and are marked by dashed lines. Adjusted R^2 values presented (in b as well).*
b) *MDM-based predictions of the lipid composition of 12 mixtures using cross validation. The mixtures were composed of different ratios of PtdCho:PS:Spg (different data points). MDM-based predictions were computed from a weighted linear sum of the MDM measurements. This linear model was fitted using leave-one-out cross validation. Predicted fractions of the three lipids (y axes) were compared to their true fraction (x axes). Dashed lines mark the identity line.*
c) *Similarity between the MDM measurements of pure lipids (x-axes) to the elements of the matrix fitted through cross validation process (y-axes). Dashed lines mark the identity line.*

Supplementary Figure 3 - Linear dependency of R1 and MTsat on MTV in the human brain.

a

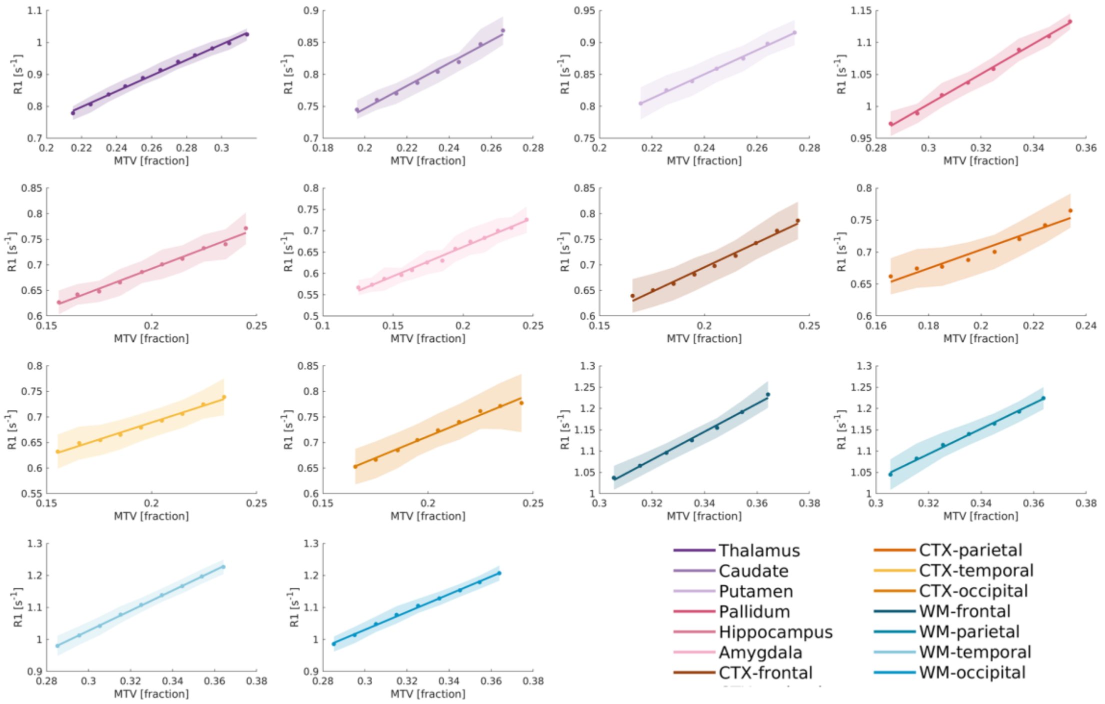

b

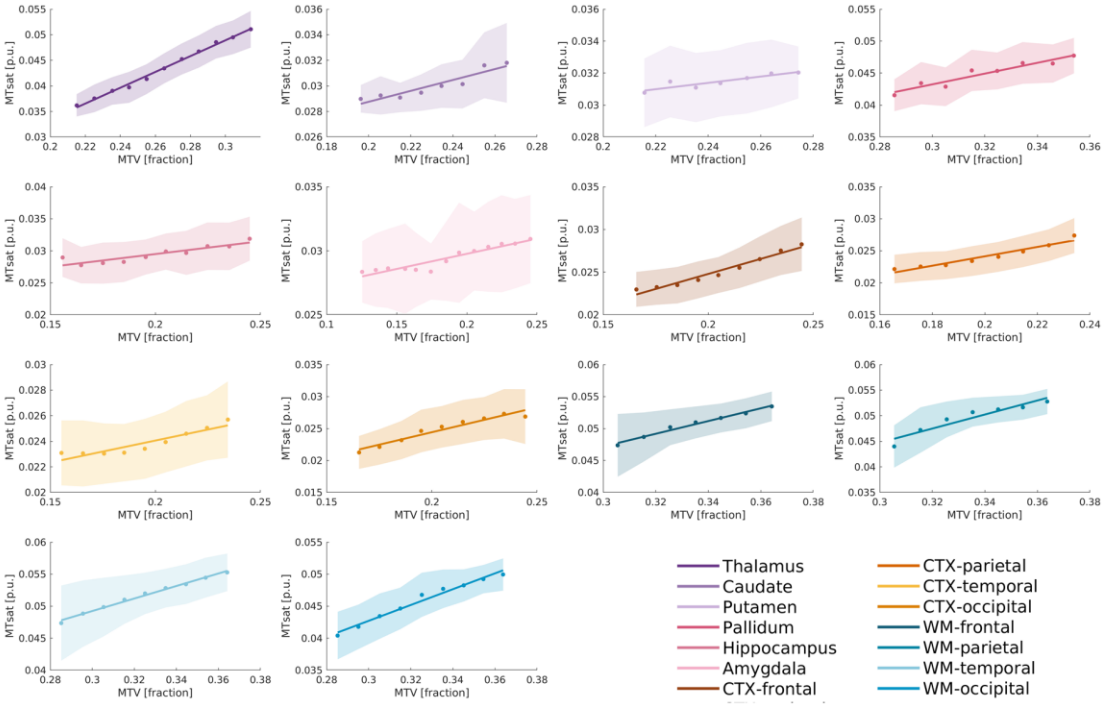

Supplementary Figure 3: Linearity of the dependency of R1 and MTsat on MTV in the human brain.
The linear relationship between R1 ***(a)*** *and MTsat* ***(b)*** *to MTV in different brain regions of a single subject. Different colors represent 14 brain regions (see legend). For each region, MTV values were pooled into bins (dots are the median of each bin; shaded area is the median absolute deviation), and a linear fit was calculated (colored lines). The slopes of the linear fit represent the MTV derivatives of R1* ***(a)*** *and MTsat* ***(b)*** *and are different for different brain areas.*

Supplementary Figure 4- Linear dependency of MD and R2 on MTV in the human brain.

a

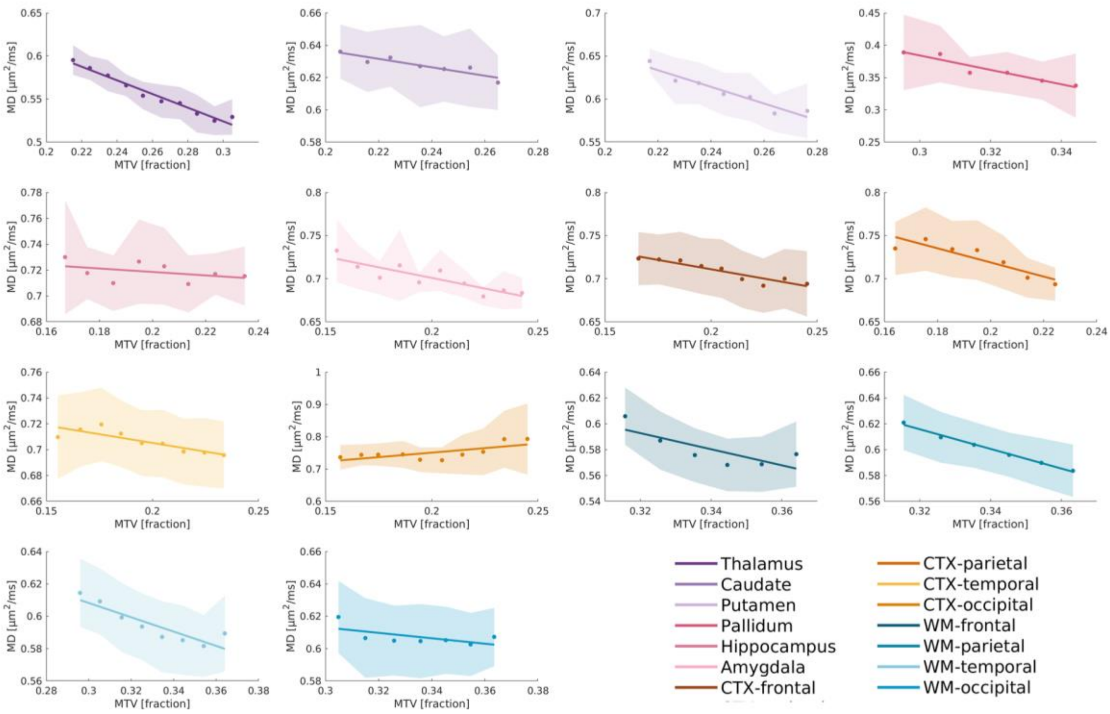

b

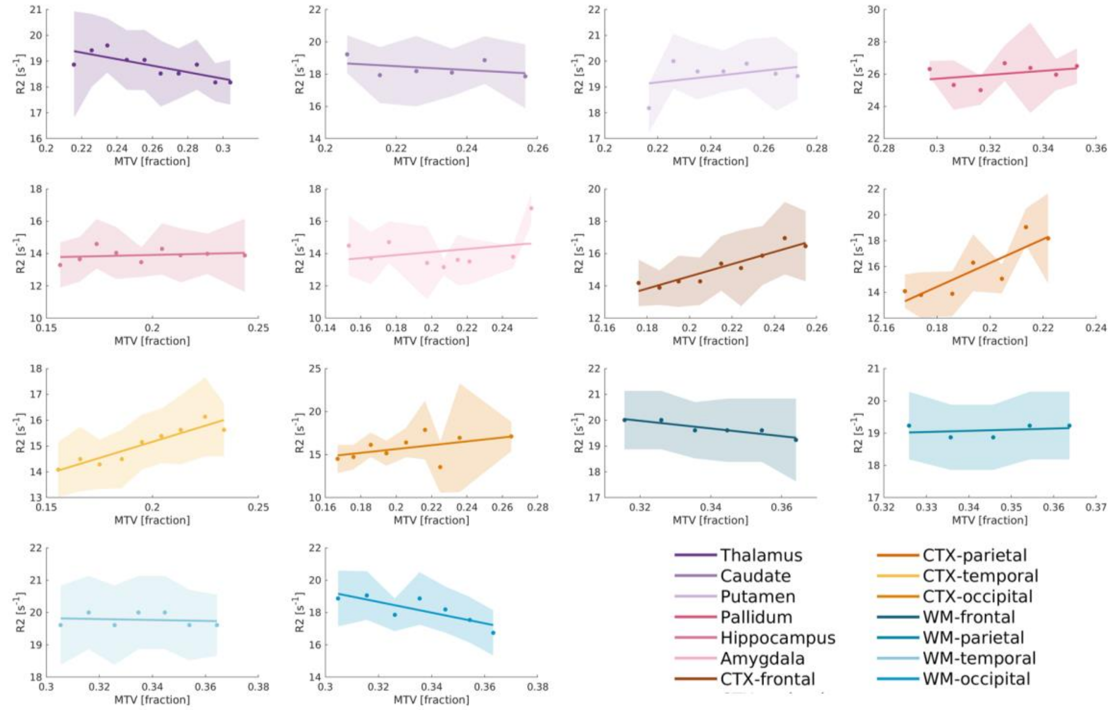

Supplementary Figure 4: Linearity of the dependency of R2 and MD on MTV in the human brain.
*The linear relationship between MD **(a)** and R2 **(b)** to MTV in different brain regions of a single subject. Different colors represent 14 brain regions (see legend). For each region, MTV values were pooled into bins (dots are the median of each bin; shaded area is the median absolute deviation), and a linear fit was calculated (colored lines). The slopes of the linear fit represent the MTV derivatives of MD **(a)** and R2 **(b)** and are different for different brain areas.*

Supplementary Figure 5 - Variance explained by the qMRI dependencies on MTV and its stability between hemispheres.

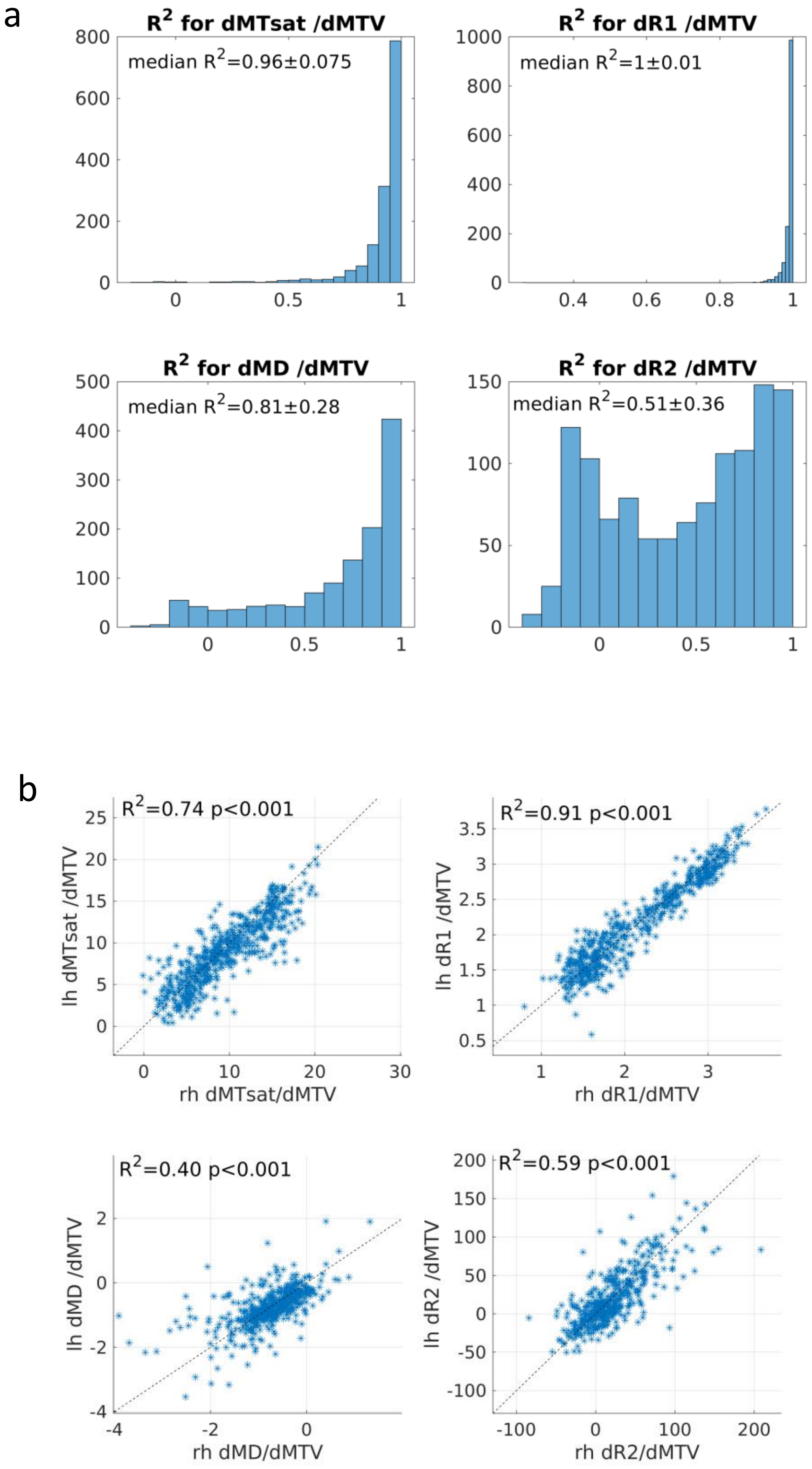

Supplementary Figure 5: Variance explained by the qMRI dependencies on MTV and its stability between hemispheres.
a) *Distributions of adjusted R^2 values acquired for the linear fit of the dependency of qMRI parameters on MTV (across brain regions and subjects). The high values for R^2 demonstrate that most of the variance in qMRI parameters can be attributed to MTV.*
b) *Agreement in the MTV derivatives of qMRI parameters between hemispheres, across brain regions and subjects. X-axis is the right hemisphere (rh) and y-axis is the left hemisphere (lh).*

Supplementary Figure 6- Distinct regional dependencies on MTV for R2 and MD.

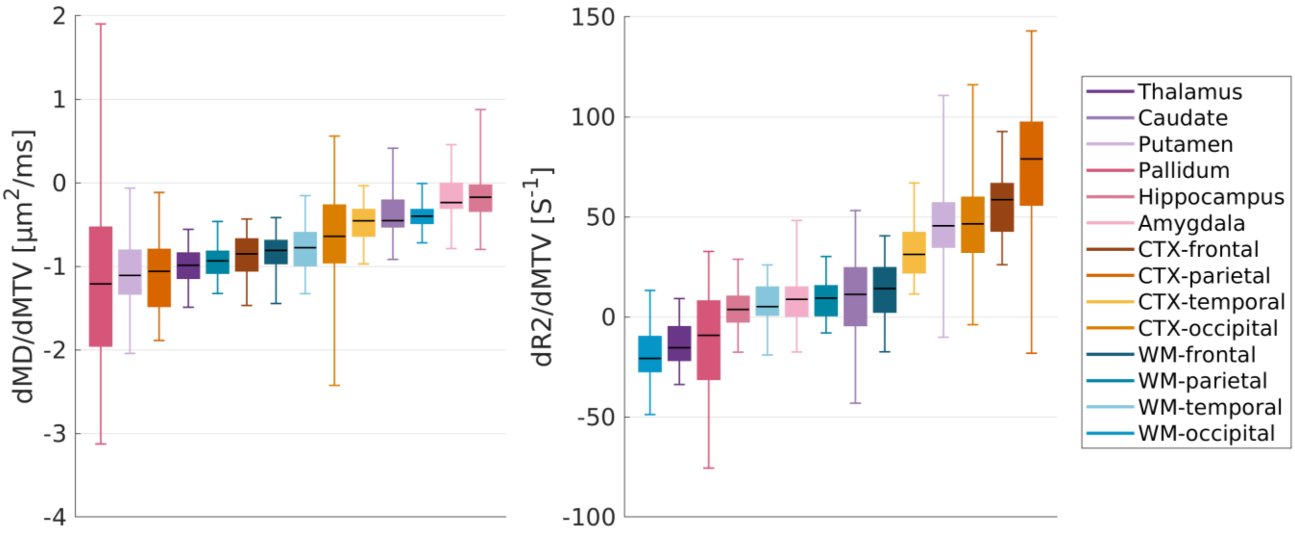

Supplementary Figure 6: Distinct regional dependencies on MTV for R2 and MD.
Variation in the MTV derivatives of MD (right) and R2 (left) across subjects (N=19 for MD, N=22 for R2). Different colors represent 14 brain regions (see legend). Edges of each box represent the 25th, and 75th percentiles, median is in black and whiskers are extreme data points. Different brain regions show different MTV derivatives of R2 and MD.

Supplementary Figure 7 - Standard qMRI parameters' variation across the brain.

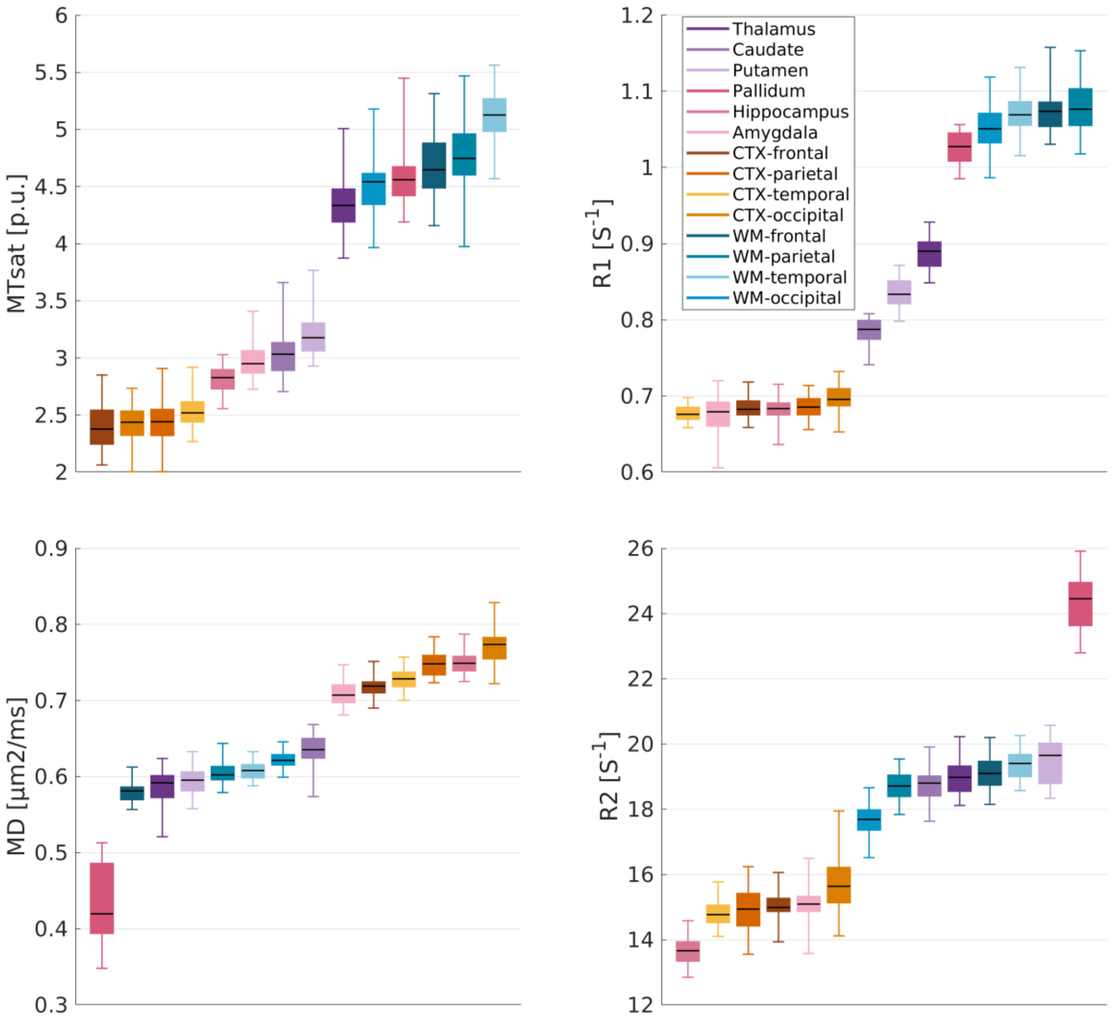

Supplementary Figure 7: Standard qMRI parameters' variation across the brain.

Variation in conventional qMRI parameters across subjects (N=23 for MTsat and R1, N=19 for MD, N=22 for R2). Different colors represent 14 brain regions (see legend). Edges of each box represent the 25th, and 75th percentiles, median is in black and whiskers are extreme data points. ***The variation between brain region in the case of conventional qMRI parameters differs from the equivalent variation in the MTV derivatives of qMRI parameters (Figure 2c).***

Supplementary Figure 8 - The MDM pipeline.

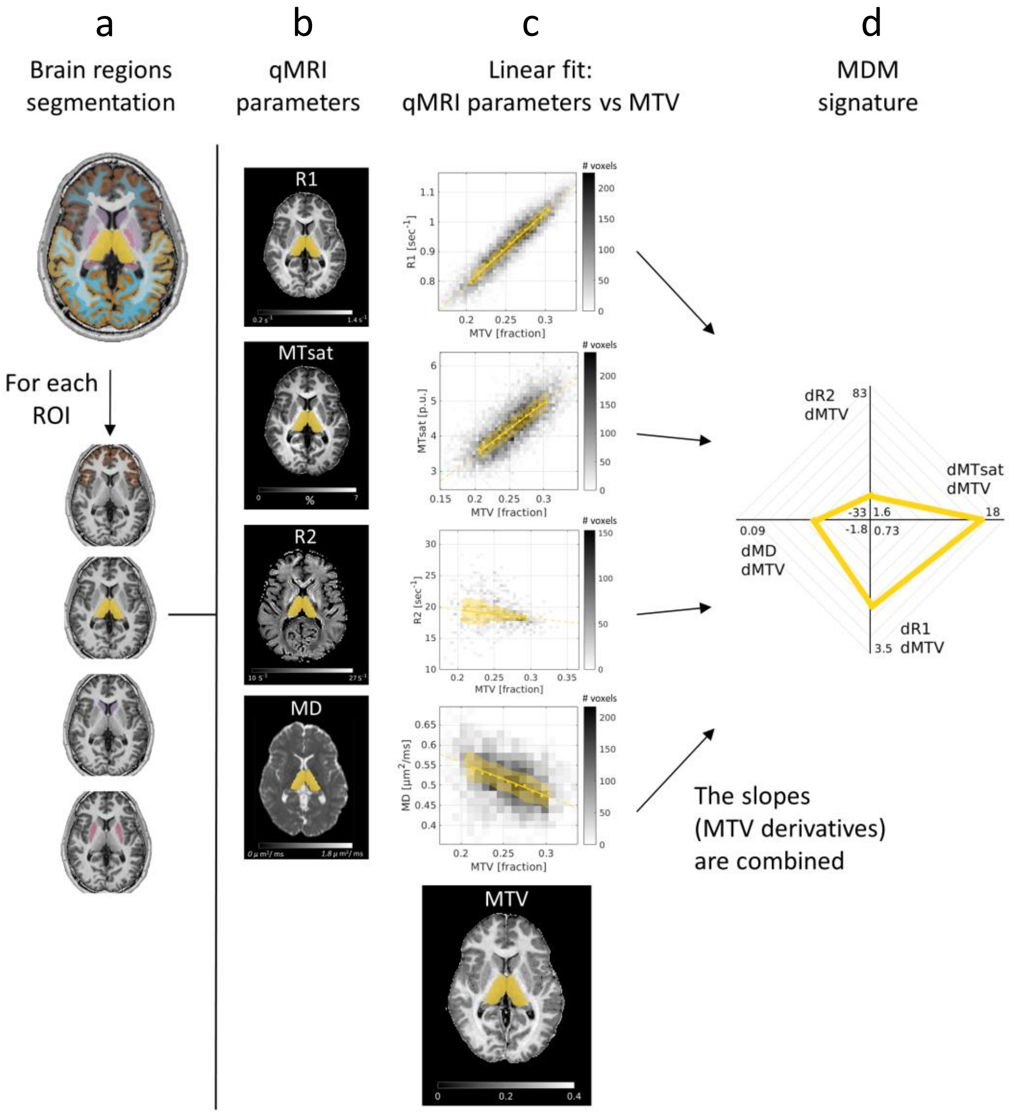

Supplementary Figure 8: The MDM pipeline.

a) *Brain regions are segmented using FreeSurfer.*

b) *Four different qMRI parameters are calculated.*

c) *For each ROI, the linear dependencies between qMRI parameters and MTV are computed; based on the 2D distribution of all the voxels in the ROI, values are pooled into bins (yellow dots are the median of each bin; shaded area is the median absolute deviation), and a linear fit is calculated (yellow lines). The slopes of the linear fit represent the MTV derivatives of qMRI parameters ("MDM measurements").*

d) *The MTV derivatives of qMRI parameters are combined to generate the MDM signature; each axis is the slope of a different qMRI parameter (R1, MTsat, R2, and MD). Colored traces extend between the MDM measurements.*

Supplementary Figure 9- Correlation of molecular features with MRI measurements.

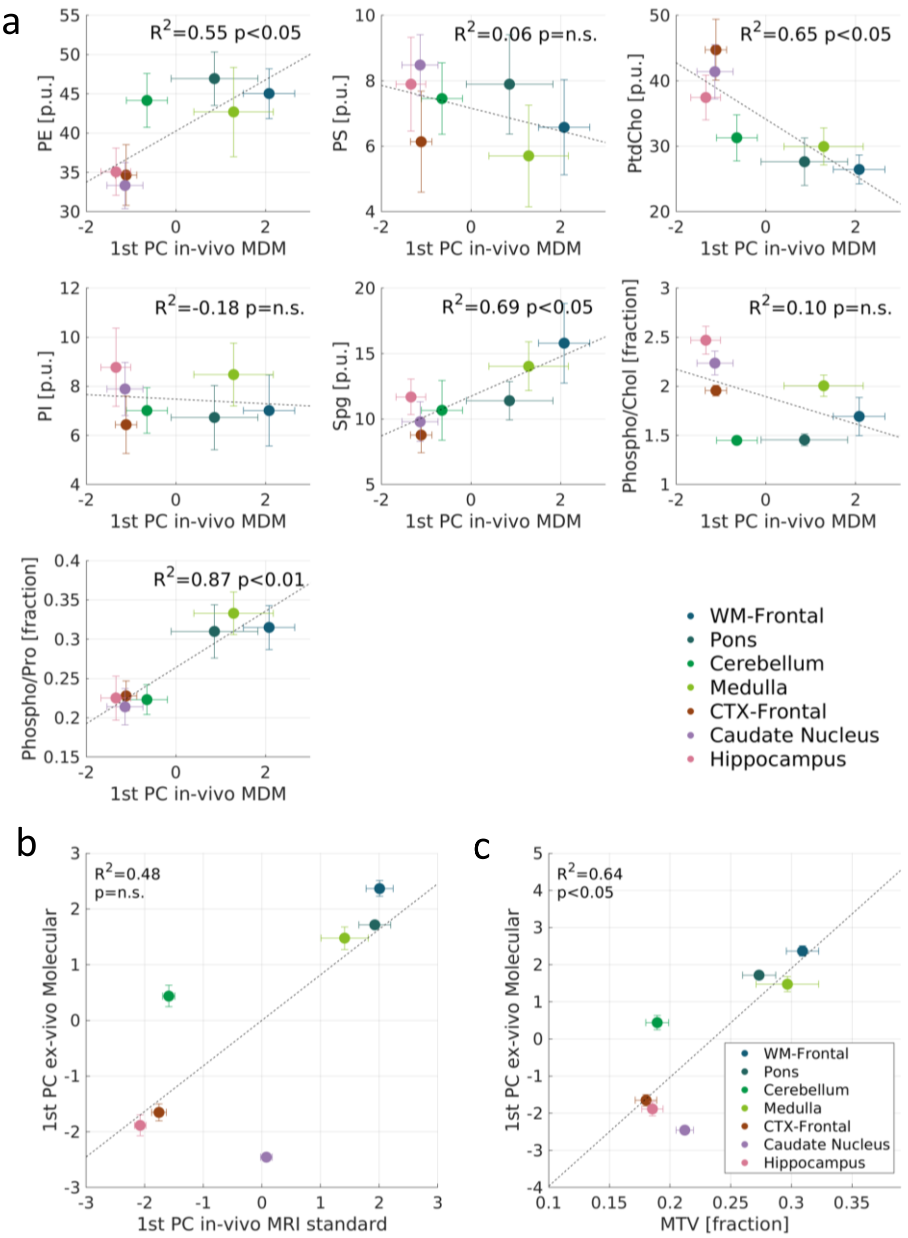

Supplementary Figure 9: Correlation of molecular features with MRI measurements.
a) *The correlation of individual lipids (derived from the literature) with the 1st principal component (PC) of in vivo MDM signatures. The lipid composition of different brain regions (y-axis, derived from the literature) is plotted against their projection on the 1st PC of standard qMRI parameters (x-axis, averaged over 19 young subjects). (phospho/pro=phospholipids/proteins, Phospho/chol=phospholipids/cholesterol). In Figure 3b the individual lipids presented here are incorporated to generate the 1st PC of* ex vivo *molecular composition. Adjusted R^2 values presented.*
b) The correlation of the molecular variability with standard qMRI parameters is lower compared to the correlation with MDM (Figure 3b). *The projection of different brain regions on the 1st PC of molecular variability (y-axis, derived from the literature) vs. their projection on the 1st PC of standard qMRI parameters (x-axis, averaged over 19 young subjects). Adjusted R^2 values presented.*
c) ***The correlation of the molecular variability with MTV is lower compared to the correlation with MDM (Figure 3b).*** *The projection of different brain regions (colored data points) on the 1st principal component (PC) of molecular variability (y-axis, derived from the literature) vs. their MTV values (x-axis, averaged over 19 young subjects). Adjusted R^2 values presented.*
Note that the cerebellum and the caudate nucleus have very different molecular compositions, as their projections on $PC^1{}_{Molecular-ex-vivo}$ are far apart. This tissue property was not detected by conventional *in vivo* qMRI methods. The two brain regions have very similar MTV (b), and their projections on $PC^1{}_{qMRI-in-vivo}$ did not capture their molecular variability (a). Nonetheless, $PC^1{}_{MDM-in-vivo}$ reflect the molecular variability of these two brain regions (Figure 3b).

Supplementary Note 4- MDM correlation with cortical gene expression patterns.

More evidence for the biological interpretability of the MDM signatures was provided by their correlation with spatial gene expression patterns throughout the cortex. We compared the cortical MDM signatures to a gene co-expression network based on a widespread survey of gene expression in the human brain undertaken by the Allen Human Brain Atlas project[2]. Nineteen modules were derived from the gene network, each comprised of a group of genes that co-varies in space. The projection of different cortical areas on the first PC of each module captures the "eigengene" of the module- the main axes of the module's variation across the brain. The eigengenes of the different modules were compared to the cortical projection on the first PC of the MDM signatures ($\mathbf{PC^1}_{\mathbf{MDM-in-vivo}}$, Figure 4a). In this case the PCA is calculated using the R1 and MTsat maps (without MD and T2 maps), to allow sufficient resolution for cortical parcellation. The correlations were corrected for multiple comparisons using the FDR method. Six out of the nineteen gene modules were significantly correlated with the first PC of MDM (adjusted R^2 ranges between 0.098 and 0.26). The two modules with the highest correlations are presented in Figure 4b (see Supplementary Figure 10a for the remaining modules). Enrichment of gene ontology categories for the six modules revealed a strong association with membranes, synapses, and neurons[2]. The membrane-associated module correlated most strongly with the MDM signatures (R^2 = 0.26, Figure 4b), thus providing data-driven support for our hypothesis regarding the sensitivity of the MDM signatures to membrane lipids. Similar analyses for standard qMRI parameters and for MTV revealed significant correlations with four modules (Supplementary Figure 10b,c), albeit to a lower degree than the correlations of MDM with the membrane and synapse modules presented in Figure 4b. To further validate that the correlation of MDM with the membrane and synapse modules is not driven by outliers we performed outlier analysis (Supplementary Figure 11). Notably, we excluded the outliers and the significant correlation of MDM and the gene modules survived.

Remarkably, among nineteen gene modules, the best correlation was with the membrane-associated module. The top connections in this module included the genes *KCNC1*, *SCN1B*, *PVALB* and *HAPLN4*[2]. These genes have shown to be highly expressed in cortical interneurons and are involved in the expression of voltage gated channels, calcium signaling and the formation of extracellular matrix. Mutations in these genes were associated with Epilepsy, Alzheimer's disease and Parkinson's disease[3–5]. Interestingly, the average expression of the membrane module was shown to increase continuously into adulthood[2]. Our results indicate that the MDM approach enhances the consistency between MRI-driven measures and histology over standard qMRI measurements. Overall, these findings suggest that the MDM signatures may be exploited in order to increase the specificity of MRI to different chemophysical components.

Supplementary Figure 10- Additional correlations of MRI with specific gene expression patterns throughout the cortex.

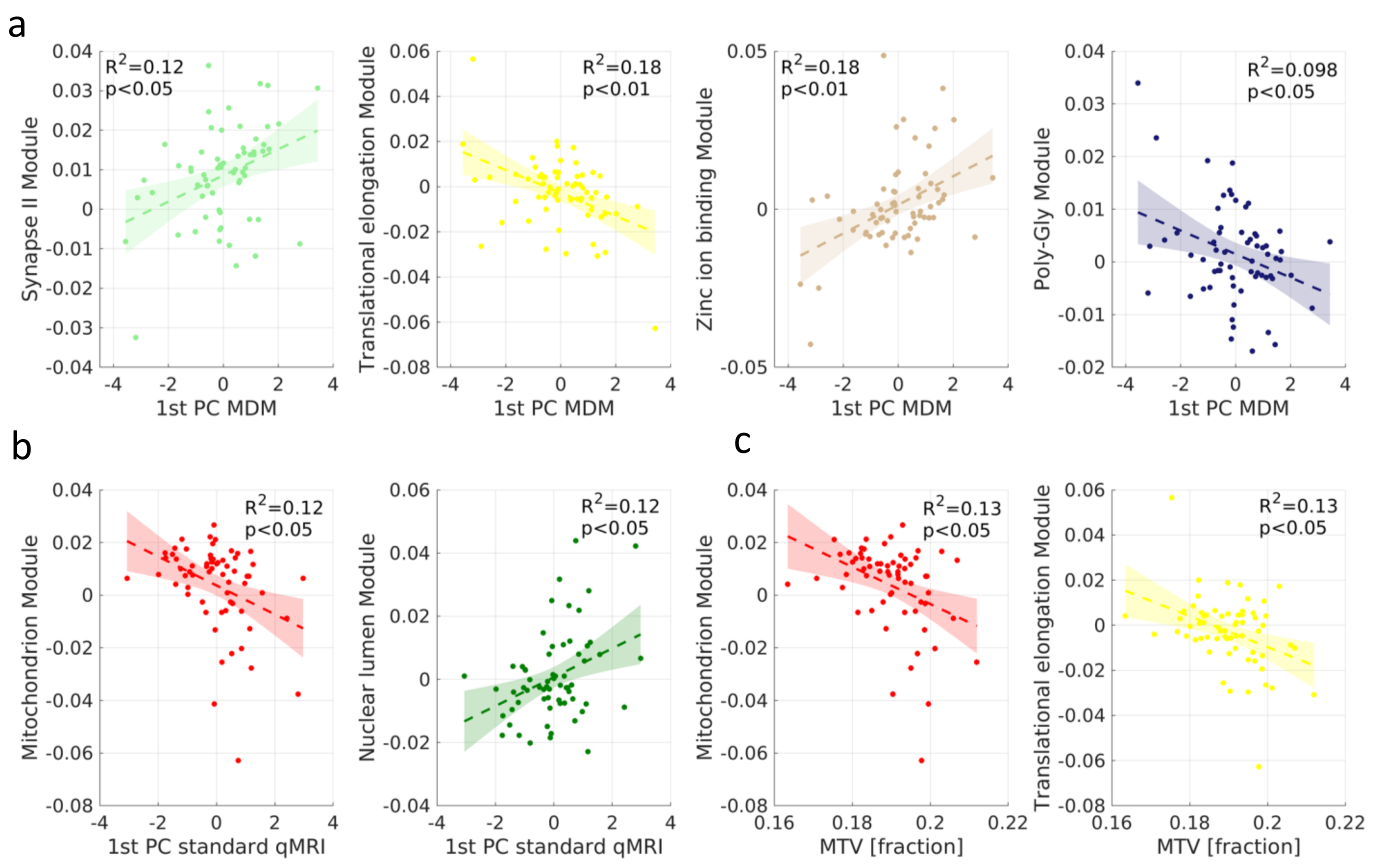

Supplementary Figure 10: Additional correlations of MRI with specific gene expression patterns throughout the cortex.
a) Four additional modules were found to significantly correlate with MDM throughout the cortex. *The projection of 64 cortical areas on the eigengenes of these gene modules (y axes) is plotted against their projection on the 1st principal component (PC) of MDM (x-axes). The synapse II module is termed "lightgreen" in Ben-David and Shifman (2012), the translational elongation module is termed "yellow", the zinc binding module is termed "tan" and the Poly-Gly module is termed "darkblue".* Adjusted R^2 values presented. P-values are for the F-test and were corrected for multiple (57) comparisons.
b) Standard qMRI parameters are less correlated with specific gene expression patterns compared to MDM. *Two modules were found to significantly correlate with the 1st PC of standard qMRI parameters (R1 and MTsat). The mitochondrion module is termed "red" in Ben-David and Shifman (2012), and the Nuclear lumen module is termed "green". The analysis was done on the young subjects.* Adjusted R^2 values presented. P-values are for the F-test and were corrected for multiple (57) comparisons.
c) MTV is less correlated with specific gene expression patterns compared to MDM. *Two modules were found to significantly correlate with MTV. The analysis was done on the young subjects.* Adjusted R^2 values presented. P-values are for the F-test and were corrected for multiple (57) comparisons.

Supplementary Figure 11- The effect of outliers on the correlation of MDM with the gene modules.

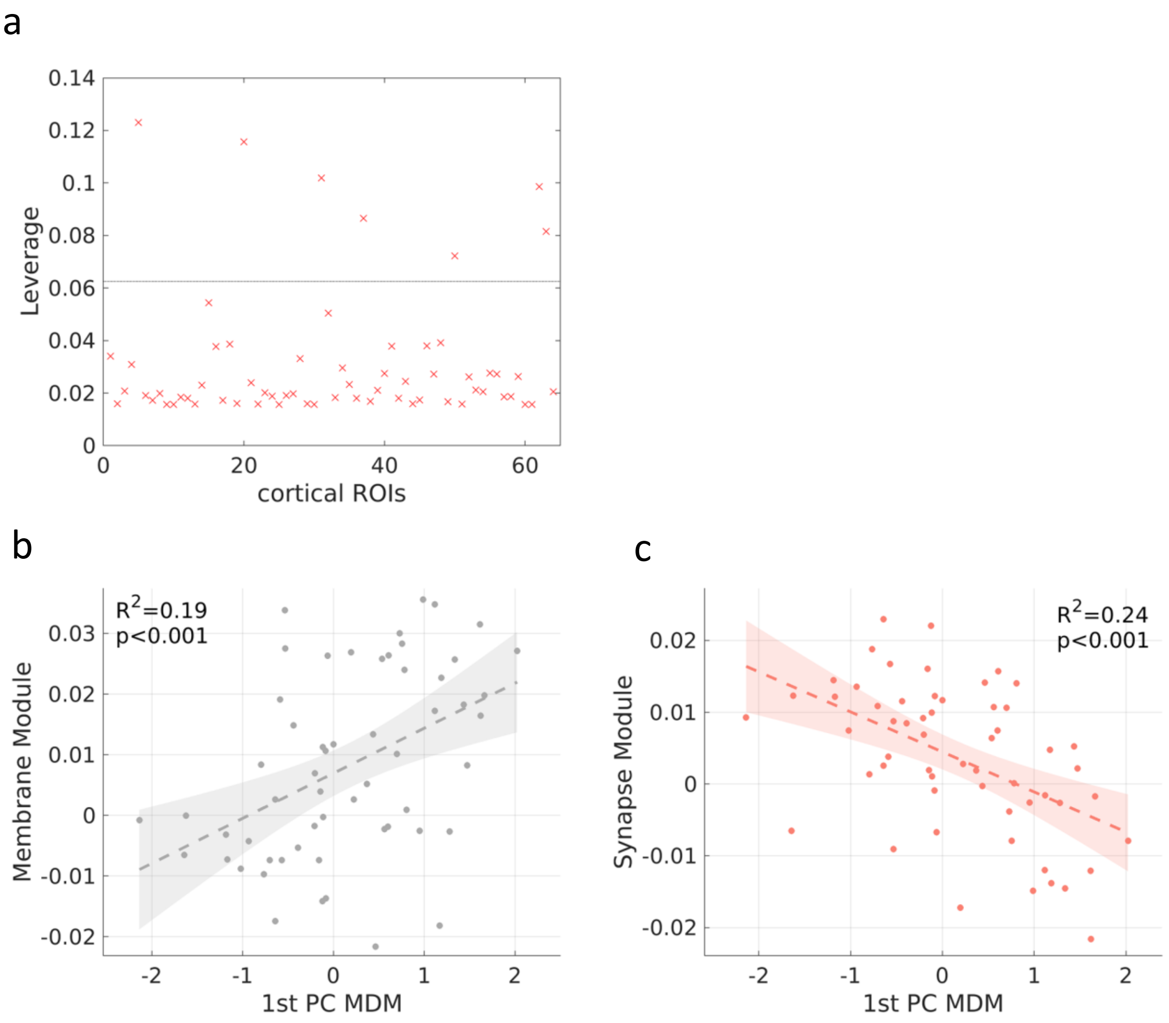

Supplementary Figure 11: The effect of outliers on the correlation of MDM with the membrane and synapse modules.

a) Identifying outliers based on high leverage. *The figure shows the leverage of the different cortical regions. Observations with high leverage values are outlying with regard to their "1st PC MDM" values, and therefore might be excessively influencing on the regression between the 1st PC of MDM and the different gene modules (shown in Figure 4b). The horizontal line represents the recommended threshold value 2p/n, where p is the number of coefficients (2), and n is the number of observations (64). Observations that exceed the threshold are identified as outliers.*

b-c) The regression between the 1st PC of MDM and two gene modules after removing the seven outliers identified in a. *The Membrane (b) and Synapse (c) modules are shown as they are the modules most correlated with the MRI measurements. The projection of cortical areas on the eigengenes of the gene modules (y-axes) is plotted against their projection on the 1st PC of in vivo MDM. The eigengenes represent the variability in the gene expression of each module through the cortex. Even after removing the outliers, this variability correlates with the variability in MDM signatures. Shaded areas represent the 95% confidence bounds for the fitted linear model. The p-values shown here are for the F-test. They survive the Bonferroni correction for multiple (60) comparisons. Adjusted R^2 values presented.*

Supplementary Figure 12- Histological and MDM analyses of the porcine brain and comparison to human.

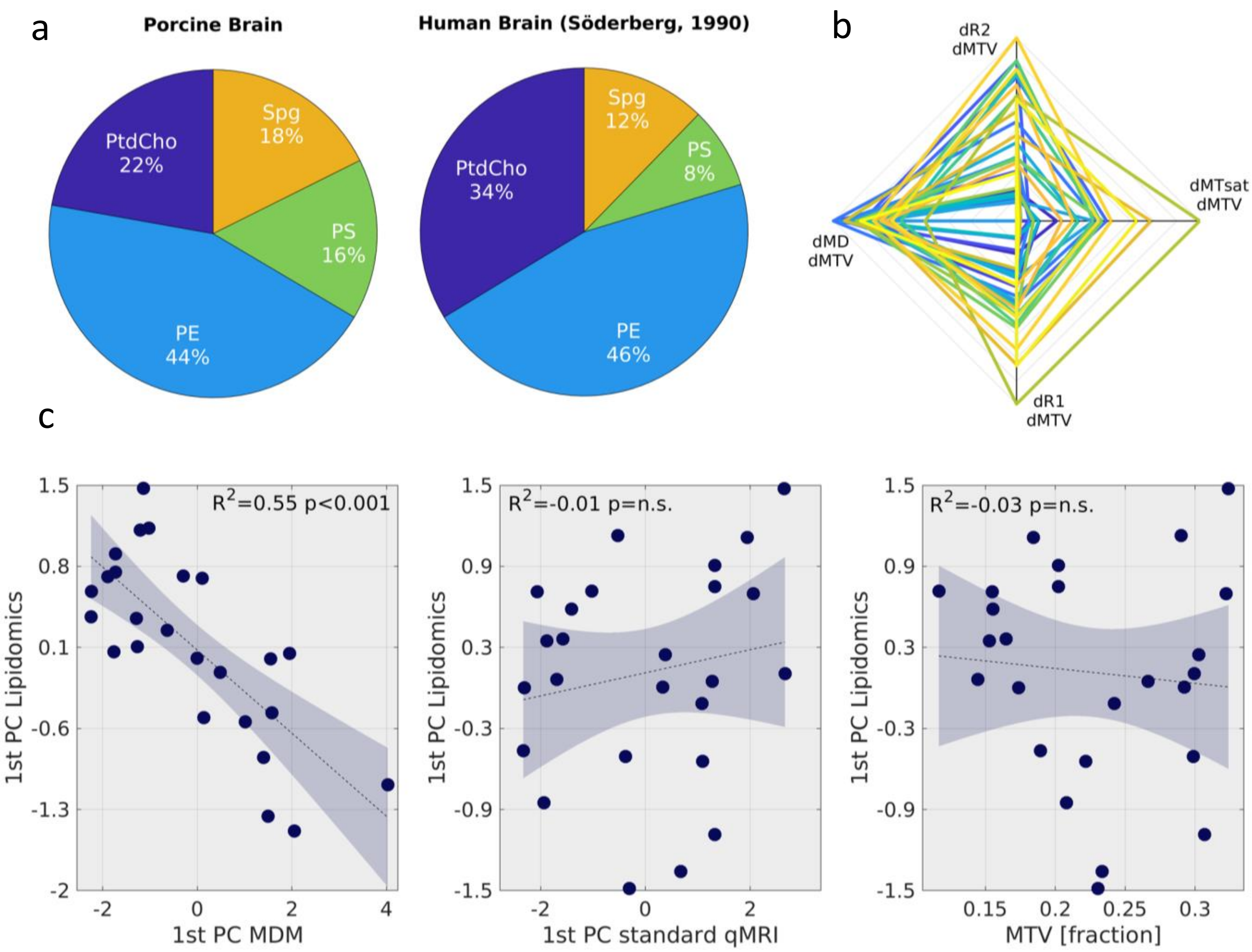

Supplementary Figure 12: Histological and MDM analyses of the porcine brain and comparison to human.

a) Phospholipid composition of the porcine and human brain.

Left: Porcine brain. The average percentage of four phospholipids as measured post-mortem using thin-layer chromatography (TLC). The percentages were calculated after taking the median of each phospholipid over 30 brain samples in two porcine brains (for raw data see Supplementary Table 2).

Right: Human brain. The average percentage of four phospholipids as measured post-mortem by Söderberg (1990). The percentages are averaged over seven brain samples. Both in the human brain and the porcine brain, PE constitute over 40% of the lipid composition.

b) Unique MDM signatures of 30 brain regions in two porcine brains. *Each signature was calculated based on a single brain ROI. Each axis is the MTV derivative ("MDM measurements") of a different qMRI parameter (R1, MTsat, R2, and MD). The range of each axis was determined based on the most extreme data points. Colored traces extend between the MDM measurements.*

c) The similarity between the molecular variability and the MDM variability in the same brain after removing outliers. *The projection of 25 brain regions (blue data points) on the 1st PC of lipidomics variability (y-axis, derived from TLC) vs. their projection on the 1st PC of MDM (Right panel, x-axis), the* 1st *PC of standard qMRI parameters (middle panel, x-axis) and MTV (left panel, x-axis). Unlike figure 5, in this case five brain regions which their projections on the* 1st *PC of lipidomics were 1.5 standard deviations away from the center were excluded from the linear regression.*

Supplementary Table 2- The lipid composition of the porcine brain.

		Neutral lipid species			Polar lipid species			
Brain	ROI	Chol	FFA	PL	PtdCho	PE	PS	Spg
brain 1	Putamen	3.3	2.11	2.62	2.79	5.72	2.85	2.6
brain 1	GM Left	2.5	2.14	2.3	2.62	5.25	2.55	2.85
brain 1	GM Right	2.72	2.23	2.78	2.49	5.77	2.31	2.18
brain 1	Striatum Left	2.85	2.23	2.22	3.08	6.26	2.3	2.21
brain 1	Striatum Right	2.75	2.66	2.24	2.4	5.85	2.27	3.15
brain 1	Brain Stem	2.95	3.46	2.32	2.17	5.76	2.28	2.21
brain 1	Thalamus Left	2.39	1.79	1.94	2.78	4.47	1.91	2.06
brain 1	Thalamus Right	2.39	1.8	2.31	2.13	5.24	1.84	1.85
brain 1	Frontal WM Right	3.41	1.93	3.24	3.33	5.08	1.95	2.05
brain 1	Frontal GM Right	2.54	1.83	2.45	3.42	5.51	1.82	1.9
brain 1	Hippocampus Right	2.63	2.07	2.31	3.57	5.7	2.38	2.3
brain 1	Hippocampus Left	2.83	1.83	2.31	3.52	5.67	2.33	2.09
brain 1	Corpus Callosum	2.89	1.57	2.46	3.21	5.58	2.23	2.23
brain 1	Frontal WM Left	2.26	1.78	2.27	2.04	3.34	1.23	1.64
brain 1	Frontal GM Left	2.74	1.83	2.84	3.22	4.98	2.28	2.21
brain 1	Cerebellum GM	2.92	2.59	2.76	2.25	4.85	1.56	2.32
brain 2	Spine	3.2	2.7	2.63	2.36	4.98	1.3	1.76
brain 2	Cerebellum WM	3.09	2.69	2.65	1.68	4.32	2.55	1.89
brain 2	Frontal GM Right	2.38	2.71	2.64	2.91	5.28	2.61	1.82
brain 2	Putamen Left	2.99	2.74	2.9	3.05	5.14	2.02	2.15
brain 2	Frontal WM Left	3.08	2.16	2.81	2	7	2.64	2.35
brain 2	Fornix & CC	2.3	4.68	2.46	2.95	5.07	1.18	1.84
brain 2	Pons	3.47	2.14	2.71	2.96	6.28	1.47	2.68
brain 2	Thalamus Left	2.66	2.42	2.35	2.56	5.48	1.32	2.13
brain 2	Hippocampus Right	1.96	4.7	2.04	2.16	3.75	1.42	1.63
brain 2	Occipital GM Left	3.13	3.51	2.77	2.78	7.63	1.83	2.65
brain 2	Subcortical Right	3.08	3.29	3.21	3.12	3.92	1.54	2.02
brain 2	Putamen Right	3.65	3.61	4.25	3.97	3.72	1.82	3.01
brain 2	Caudate Right	2.8	3.09	3.1	2.44	3.15	1.23	1.55
brain 2	Occipital WM Right	3.72	3.13	3.37	2.93	6.91	1.58	2.87

Supplementary Table 2: The lipid composition of the porcine brain.
Post-mortem lipidomics analysis of two porcine brains was done using thin-layer chromatography (TLC). Values in the table are in 10^9XDigital Light Unit (DLU) as extracted from the TLC plates using OptiQuant™ software. These values were used in figure 5 to calculate the 1st principal component of lipidomics. Chol=Cholesterol, FFA= free fatty acids, PL= phospholipids, PtdCho= Phosphatidylcholine, PE= Phosphatidylethanolamine, PS= Phosphatidylserine, Spg= Sphingomyelin.

Supplementary Figure 13 - aging-related changes in MDM signature

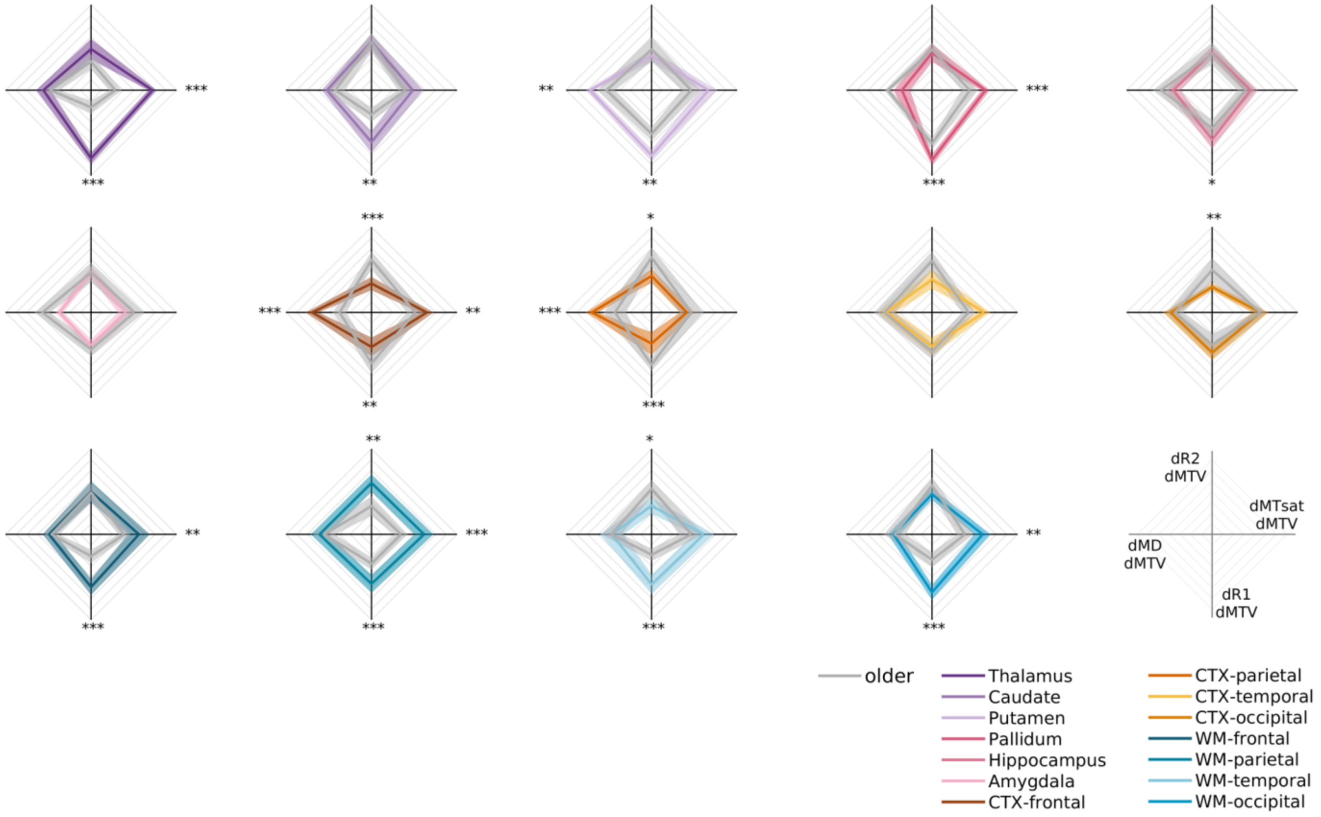

Supplementary Figure 13: aging-related changes in MDM signatures.
*Comparison of the MDM signatures of 18 older adults (aged 67±6 years, marked in gray) and 23 younger adults (aged 27±2 years, marked with different colors). Multidimensional aging-related changes revealed by the MDM approach are presented for different brain regions (young subjects are represented by different colors; older subjects are in gray). Each axis is the MTV derivative of a different qMRI parameter. The range of each axis was determined by the 5 and 95 Percentiles. Traces extends between these derivatives; shaded areas represent the variation across subjects. The statistical significance of the differences between the groups was estimated using a two-sample t-test and was corrected for multiple comparisons using the FDR method. * P < 0.05; ** P < 0.01; *** P < 0.001.*

Supplementary Figure 14 - separating molecular and water related contributions.

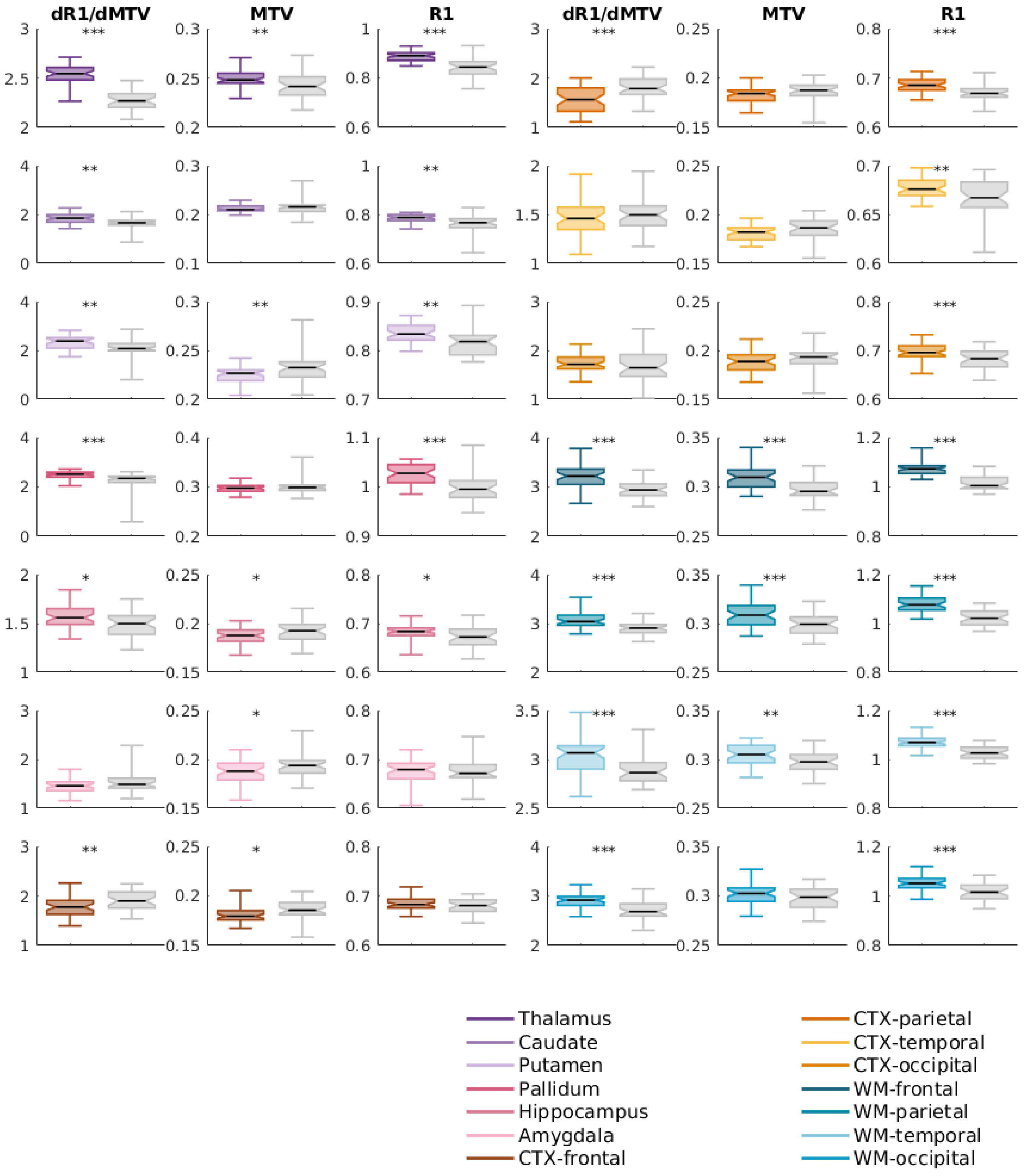

Supplementary Figure 14: separating R1 measurement to its molecular and water related constituents.
*Comparison of MRI-driven measurements of 18 older adults (aged 67±6 years, marked in gray) and 23 younger adults (aged 27±2 years, marked with different colors) in different brain regions (see legend). Aging-related changes revealed by R1 measurement are presented in the R1 columns. The separation of chemophysical and water related constituents estimated by the MTV derivative and MTV respectively is shown in the dR1/dMTV and MTV columns. The statistical significance of the differences between the groups was estimated using a two-sample t-test and was corrected for multiple comparisons using the FDR method. * P < 0.05;** P < 0.01; *** P < 0.001.*

Supplementary Figure 15- separating molecular and water related contributions.

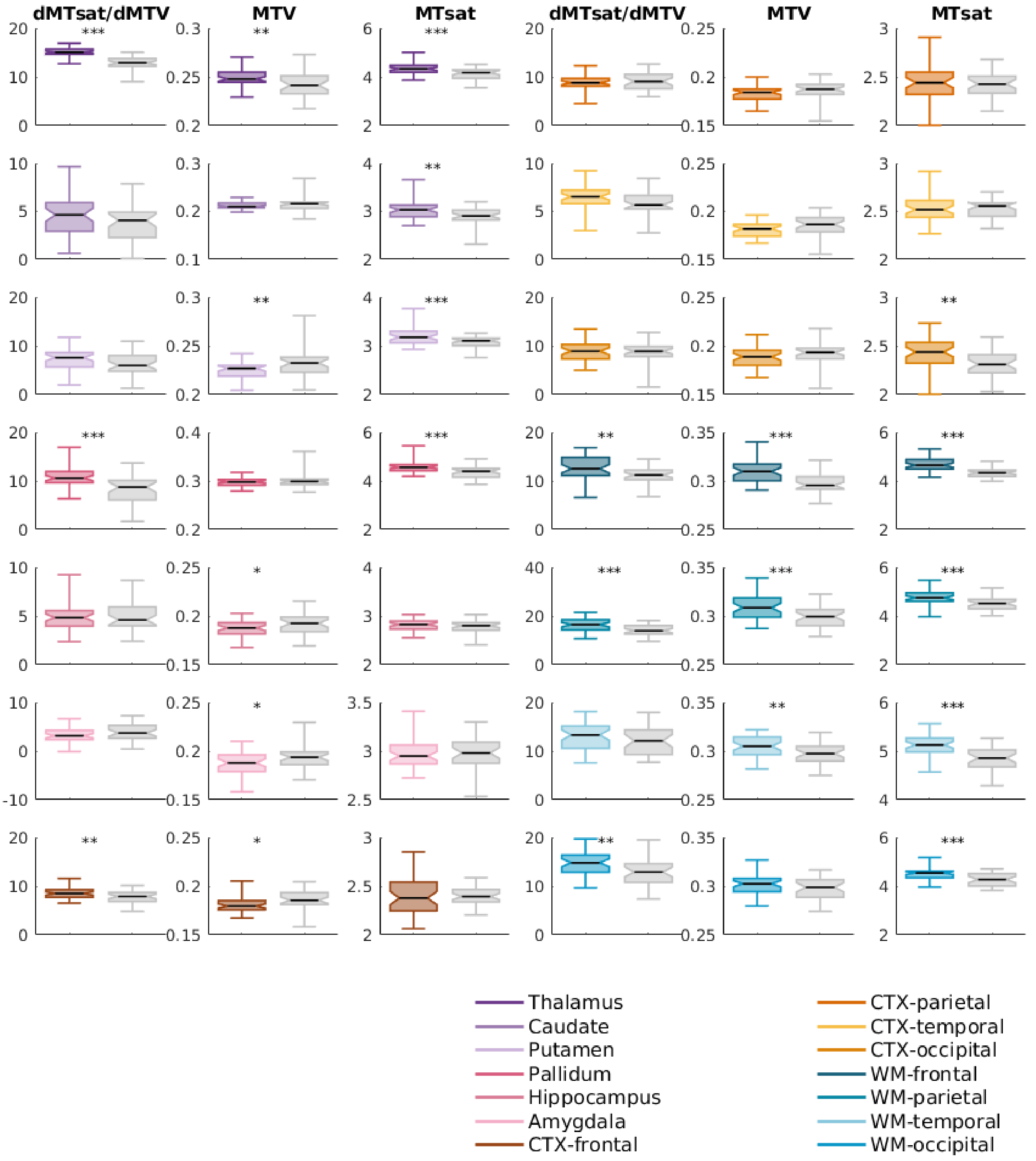

Supplementary Figure 15: separating MTsat measurement to its molecular and water related contributions. *Comparison of MRI-driven measurements of 18 older adults (aged 67±6 years, marked in gray) and 23 younger adults (aged 27±2 years, marked with different colors) in different brain regions (see legend). Aging-related changes revealed by MTsat measurement are presented in the MTsat columns. The separation of chemophysical and water related contributions estimated by the MTV derivative and MTV respectively is shown in the dMTsat/dMTV and MTV columns. The statistical significance of the differences between the groups was estimated using a two-sample t-test and was corrected for multiple comparisons using the FDR method. * P < 0.05; ** P < 0.01; *** P < 0.001.*

Supplementary Figure 16- separating molecular and water related contributions.

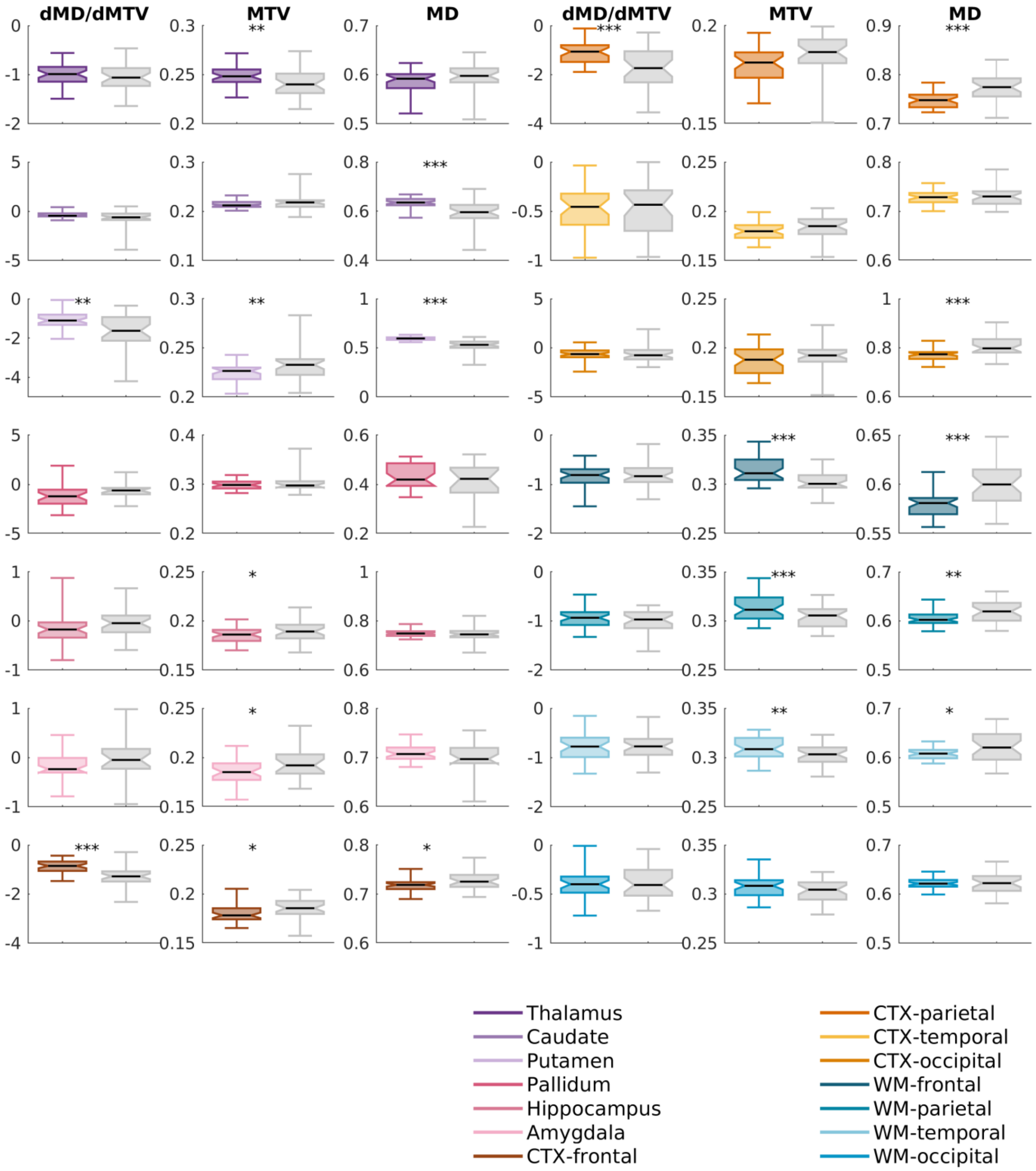

Supplementary Figure 16: separating MD measurement to its molecular and water related contributions. *Comparison of MRI-driven measurements of 17 older adults (aged 68±5 years, marked in gray) and 19 younger adults (aged 27±2 years, marked with different colors) in different brain regions (see legend). Aging-related changes revealed by MD measurement are presented in the MD columns. The separation of chemophysical and water related contributions estimated by the MTV derivative and MTV respectively is shown in the dMD/dMTV and MTV columns. The statistical significance of the differences between the groups was estimated using a two-sample t-test and was corrected for multiple comparisons using the FDR method.*

** P < 0.05;*

*** P < 0.01;*

**** P < 0.001.*

Supplementary Figure 17- separating molecular and water related contributions.

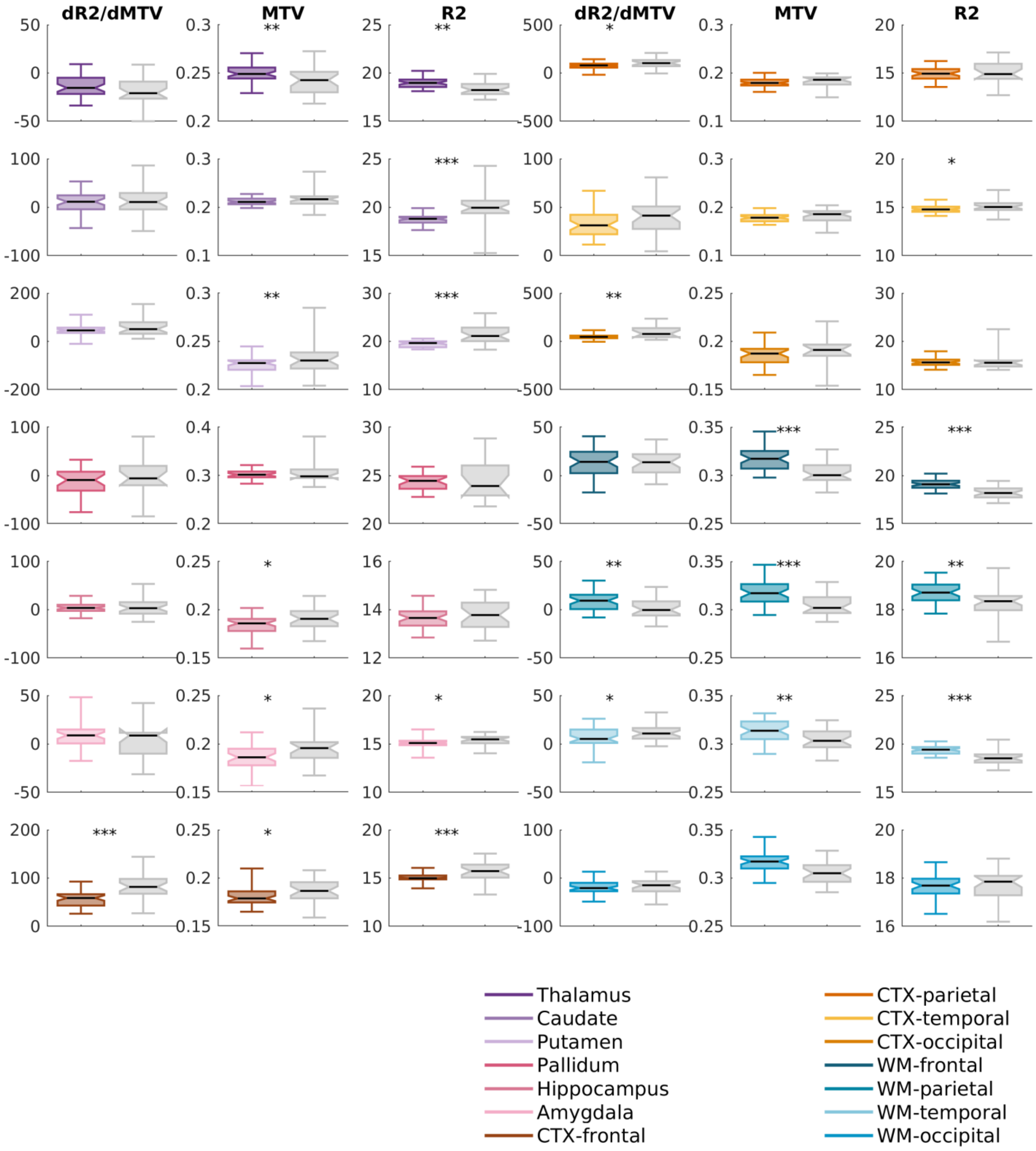

Supplementary Figure 17: separating R2 measurement to its molecular and water related contributions. *Comparison of MRI-driven measurements of 15 older adults (aged 69±5 years, marked in gray) and 22 younger adults (aged 27±2 years, marked with different colors) in different brain regions (see legend). Aging-related changes revealed by R2 measurement are presented in the R2 columns. The separation of chemophysical and water related contributions estimated by the MTV derivative and MTV respectively is shown in the dR2/dMTV and MTV columns. The statistical significance of the differences between the groups was estimated using a two-sample t-test and was corrected for multiple comparisons using the FDR method. * P < 0.05;** P < 0.01;*** P < 0.001.*

Supplementary Figure 18- aging related changes revealed by the 1st principle component of MDM.

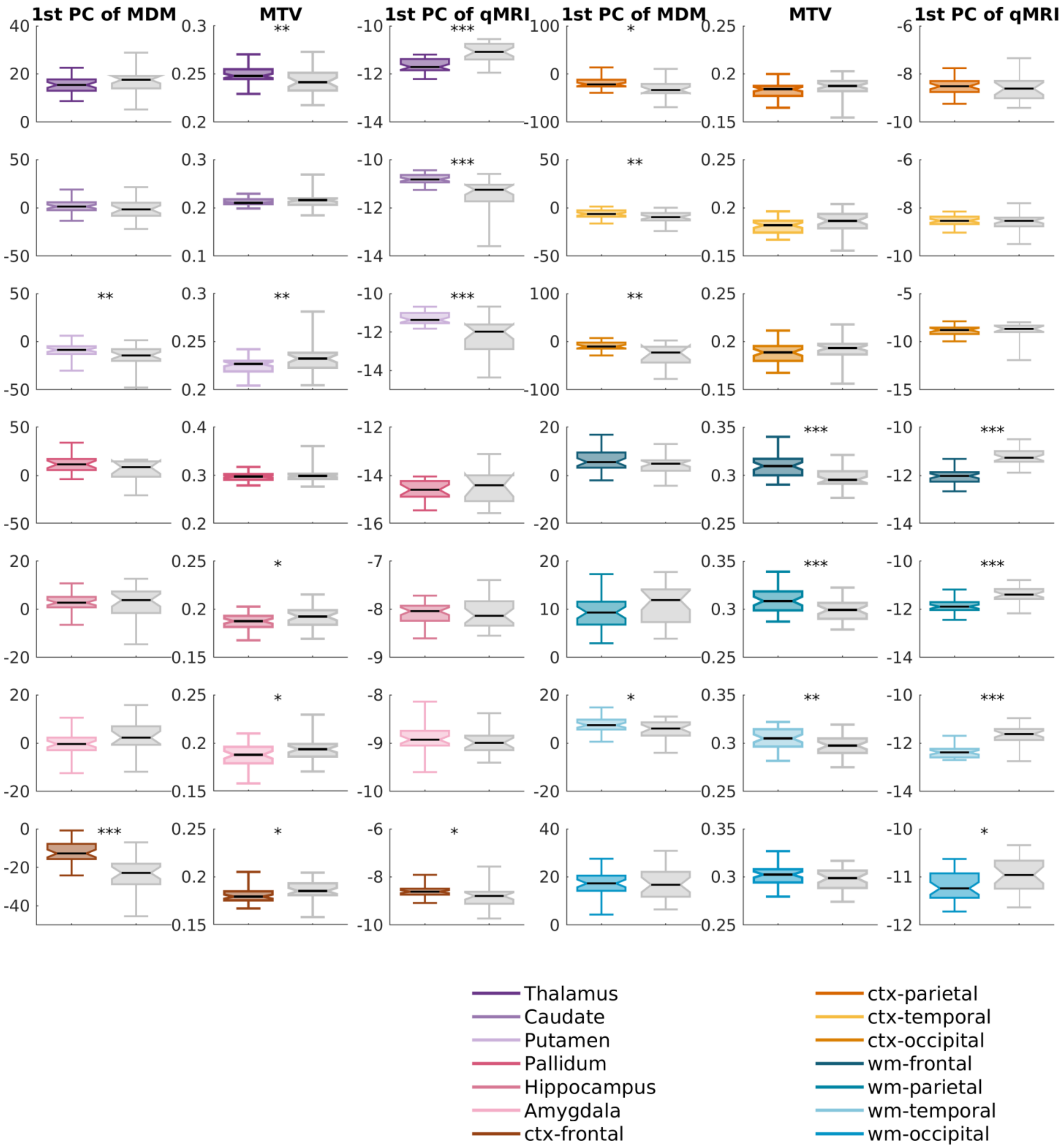

Supplementary Figure 18: aging related changes revealed by the 1st principal component of MDM.
Comparison of MRI-driven measurements of older adults (aged 69±5 years, marked in gray) and younger adults (aged 27±2 years, marked with different colors) in different brain regions (see legend). Aging-related changes revealed by the 1st principal component (PC) of MDM are presented in the left columns. The aging related changes revealed by MTV are in the middle columns. The aging related changes reveald by the 1st PC of standard qMRI parameters are in the right columns. PCA was computed after taking the avarage of each measurment for the two groups. The statistical significance of the differences between the groups was estimated using a two-sample t-test and was corrected for multiple comparisons using the FDR method.
** $P < 0.05$;** $P < 0.01$;*** $P < 0.001$.*

Supplementary Note 5- The effect of iron content on MDM signatures.
We find that the MDM signatures correlate with the molecular composition of the human brain (Figure 3). However, R1 and R2, which are used to generate the MDM signatures, are not sensitive only to the molecular environment and the water content but also to the iron content [6,7]. Iron is an important component of human brain tissue. Iron seems to accumulate in different brain regions during aging, and in a variety of CNS disorders [8]. We validated that the aging-related changes observed in the MTV derivative of R1 and R2 cannot be fully explained by alterations in the iron content with age. For this aim we used R2*, as it was shown to be a good surrogate marker of iron concentration [7]. We compared R2* values between the two age groups on a subset of our subjects (15 older adults and 17 younger adults) for which we acquired R2* map.

Supplementary Figure 19 shows the age-related differences in R2* in different brain regions. In the parietal white matter, we did find significant age-related changes in the MTV derivative of R1 and in MTV (Figure 6a). However, there were no significant age-related changes in R2* (Supplementary Figure 19). We therefore assume that the aging-related changes we found in the parietal white matter are not related to alterations in iron content. In other white matter regions, there were no R2* differences between the age groups as well (Supplementary Figure 19).

In the thalamus, we found a significant age-related change in the MTV derivative of R1 (Figure 6b). However, there was no significant change in the thalamus R2* values between the age groups (Supplementary Figure 19). This indicates that the age-related changes in the thalamus revealed by the MDM method are probably not related to iron effects. In contrast, for the caudate, putamen, hippocampus and pallidum, we found significant age-related changes in the MTV derivatives of R1, along with significant changes in R2* values (Supplementary Figure 19).
In the parietal cortex, we found a significant age-related change in the MTV derivative of R1 (Figure 6c). However, in all cortical areas we found a significant change in iron content with age, as estimated by R2* (Supplementary Figure 19). This implies that some of the age-related changes in R1 can be explained by alterations in the iron content. Notably, in the frontal cortex we also found significant aging-related change in the MTsat dependency on MTV. As iron is less likely to affect MTsat measurements, it is probable that there are molecular alterations in this brain regions that cannot be explained by iron.

The MTV derivative of R2 revealed significant aging-related changes in three grey matter cortical regions (frontal cortex, parietal cortex and occipital cortex). These three brain regions also showed significant changes in R2* values with age.

The R2* analysis demonstrates that in some brain regions aging-related changes in the MTV derivative coincide with aging related changes in the iron content. In other brain regions aging-related changes in these two measures don't overlap. Therefore, the differences between the age groups revealed by MDM measurements probably incorporate some iron-related changes. However, these iron-related changes do not fully account for our findings. Hence MDM and R2* provide complementary information.

Supplementary Figure 19- Iron-related changes between the two age groups.

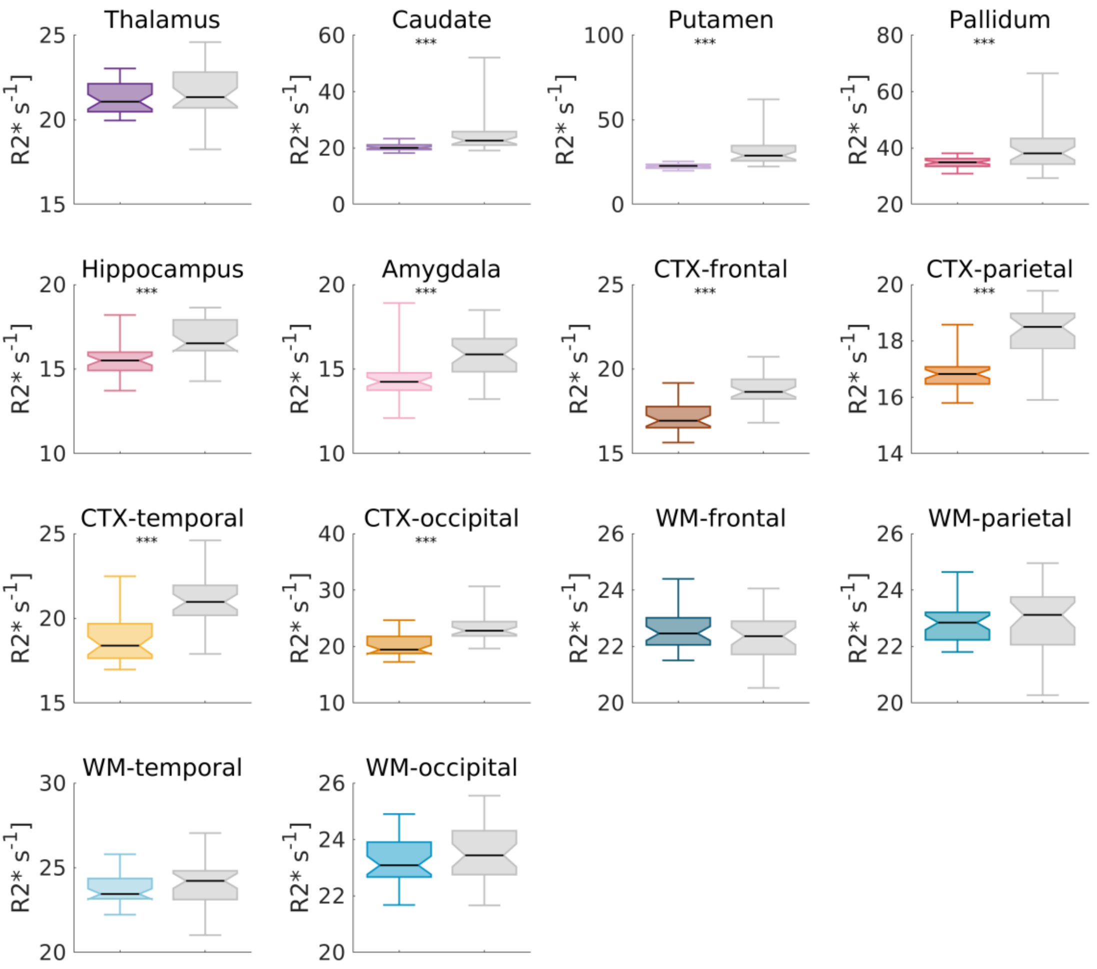

Supplementary Figure 19: Iron-related changes between the two age groups.
Comparison of R2 measurements which are related to iron content in 15 older adults (marked in gray) and 17 younger adults (marked with different colors) in different brain regions (see legend).*
*The statistical significance of the differences between the groups was estimated using a two-sample t-test and was corrected for multiple comparisons using the FDR method. * P < 0.05; ** P < 0.01; *** P < 0.001.*

Supplementary Note 6- Correction for R2* weighting in MTV estimation.

While our MTV estimation in the human brain was computed from MRI scans with relatively short TE (3.34 ms), it could still incorporate some R2* weighting.

In order to validate that our MTV estimates are not affected by this R2*-contamination, we corrected our MTV estimation for R2* in a subset of our subjects (18 old, 17 young, for further details see "R2* correction for MTV" in the methods).

Supplementary Figure 20 shows the variation across subjects in the regional MTV derivatives of R1, MTsat, MD and R2 after this correction. Compared to the uncorrected case (Figure 2c & Supplementary Figure 6) we did observe some minor differences. Nonetheless, the general regional trend was preserved.

The significant aging related changes in the MTV derivatives of R1 and MTsat we presented in Figure 6-7 survived the R2* correction (Supplementary Figure 21-22). However, after correcting MTV for R2* we could no longer identify the aging-related increase in MTV values of the frontal cortex and the hippocampus.

Across all brain regions except for the frontal cortex and the amygdala, the significant changes in the MTV derivative of R1 survived the R2* correction (Supplementary Figure 23). For the MTV derivative of MTsat, significant age-related changes in the putamen diminished after the R2* correction, while the aging-related changes in the other brain regions remained (Supplementary Figure 24).

The significance of the aging-related changes observed in the MTV derivative of MD in the temporal cortex (Supplementary Figure 25) and in the MTV derivative of R2 in the thalamus and temporal cortex did not survive the correction (Supplementary Figure 26). Interestingly, the R2* correction revealed significant aging-related changes in some brain regions that were not observed previously.

Importantly, the agreement between MDM and histology presented in figure 3 was still valid after MTV was corrected for R2* (Supplementary Figure 27).

In conclusion, the R2* correction did not dramatically affect our results. Importantly, the correction introduced additional noise to the MTV maps, as we divided by the R2* contribution to the signal (see methods). Moreover, the comparison between the corrected and uncorrected MTV was done on 35 of our 41 subjects.

Supplementary Figure 20- Distinct regional dependencies on MTV following R2* correction.

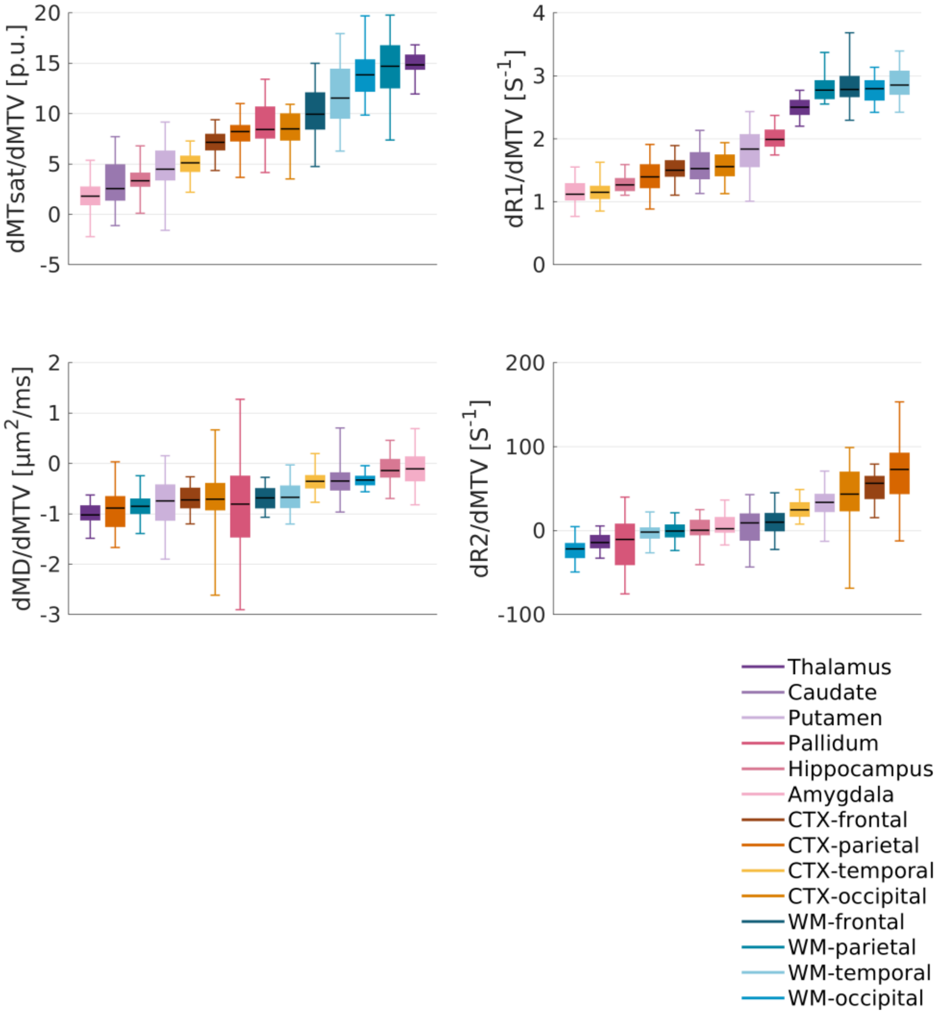

Supplementary Figure 20: Distinct regional dependencies on MTV following R2* correction.

Variation in the MTV derivatives of R1 (A), MTsat (B), R2 (C) and MD (D) across subjects for the case in which MTV was corrected for R2 (MTV_C). Different colors represent 14 brain regions (see legend). Edges of each box represent the 25th, and 75th percentiles, median is in black and whiskers are extreme data points. The regional pattern of MTV dependencies is relatively conserved after the correction, compared to the uncorrected case (Figure 2c & Supplementary Figure 6).*

Supplementary Figure 21- Aging-related changes revealed by the R1 dependency on MTV– R2* correction.

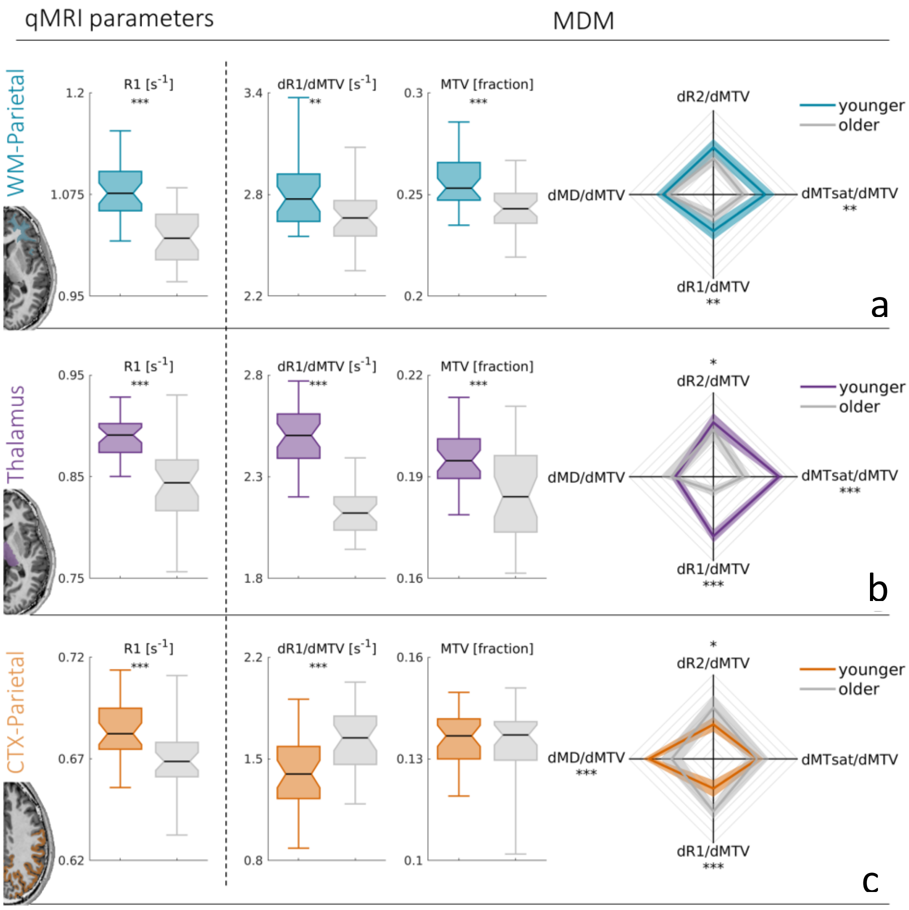

Supplementary Figure 21: Aging-related changes revealed by the R1 dependency on MTV – R2 correction.*
Comparison of MRI-driven measurements of 18 older adults (marked in gray) and 17 younger adults (marked with different colors) when MTV was corrected for R2. Aging-related changes in the parietal white matter **(a)**, the thalamus **(b)** and the parietal cortex **(c)** are similar to the uncorrected case (Figure 6). Aging-related changes revealed by standard qMRI parameters are presented in the left column. The separation to chemophysical and water related constituents estimated by the MTV derivative and MTV respectively is shown in the middle columns. Multidimensional aging-related changes revealed by the MDM approach are presented in the right column. Each axis represents the MTV derivative of a different qMRI parameter. Axes limits were set to the 5 and 95 percentiles. Traces extends between these derivatives, shaded areas represent the variation across subjects. The statistical significance of the differences between the groups was estimated using a two-sample t-test and was corrected for multiple comparisons using the FDR method.*
** $P < 0.05$; ** $P < 0.01$; *** $P < 0.001$.*

Supplementary Figure 22- Aging-related changes revealed by the MTsat dependency on MTV– R2* correction.

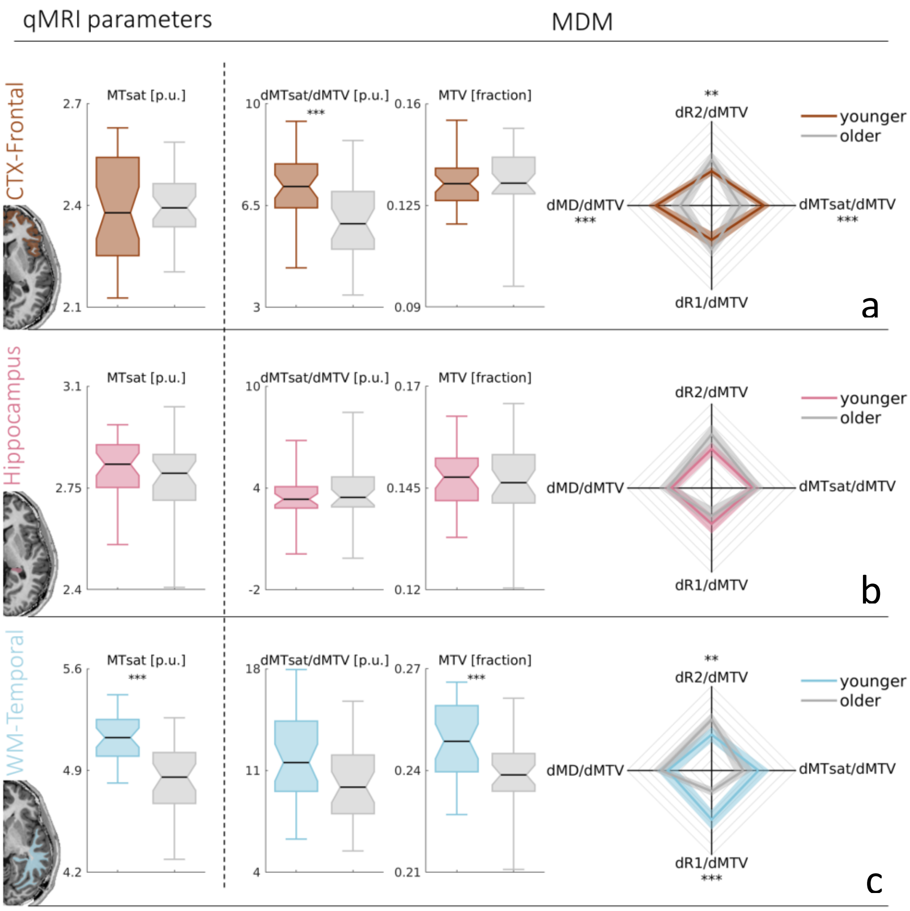

Supplementary Figure 22: Aging-related changes revealed by the MTsat dependency on MTV – R2 correction.*
Comparison of MRI-driven measurements of 18 older adults (marked in gray) and 17 younger adults (marked with different colors) when MTV was corrected for R2. Aging-related changes in the frontal cortex* ***(a)****, the hippocampus* ***(b)*** *and the temporal white-matter* ***(c)*** *are similar to the uncorrected case (Figure 7). Aging-related changes revealed by standard qMRI parameters are presented in the left column. The separation to chemophysical and water related constituents estimated by the MTV derivative and MTV respectively is shown in the middle columns. Multidimensional aging-related changes revealed by the MDM approach are presented in the right column. Each axis represents the MTV derivative of a different qMRI parameter. Axes limits were set to the 5 and 95 percentiles. Traces extends between these derivatives, shaded areas represent the variation across subjects. The statistical significance of the differences between the groups was estimated using a two-sample t-test and was corrected for multiple comparisons using the FDR method.*
** P < 0.05; ** P < 0.01; *** P < 0.001.*

Supplementary Figure 23- Aging-related changes in R1 revealed by the MDM approach – R2* correction.

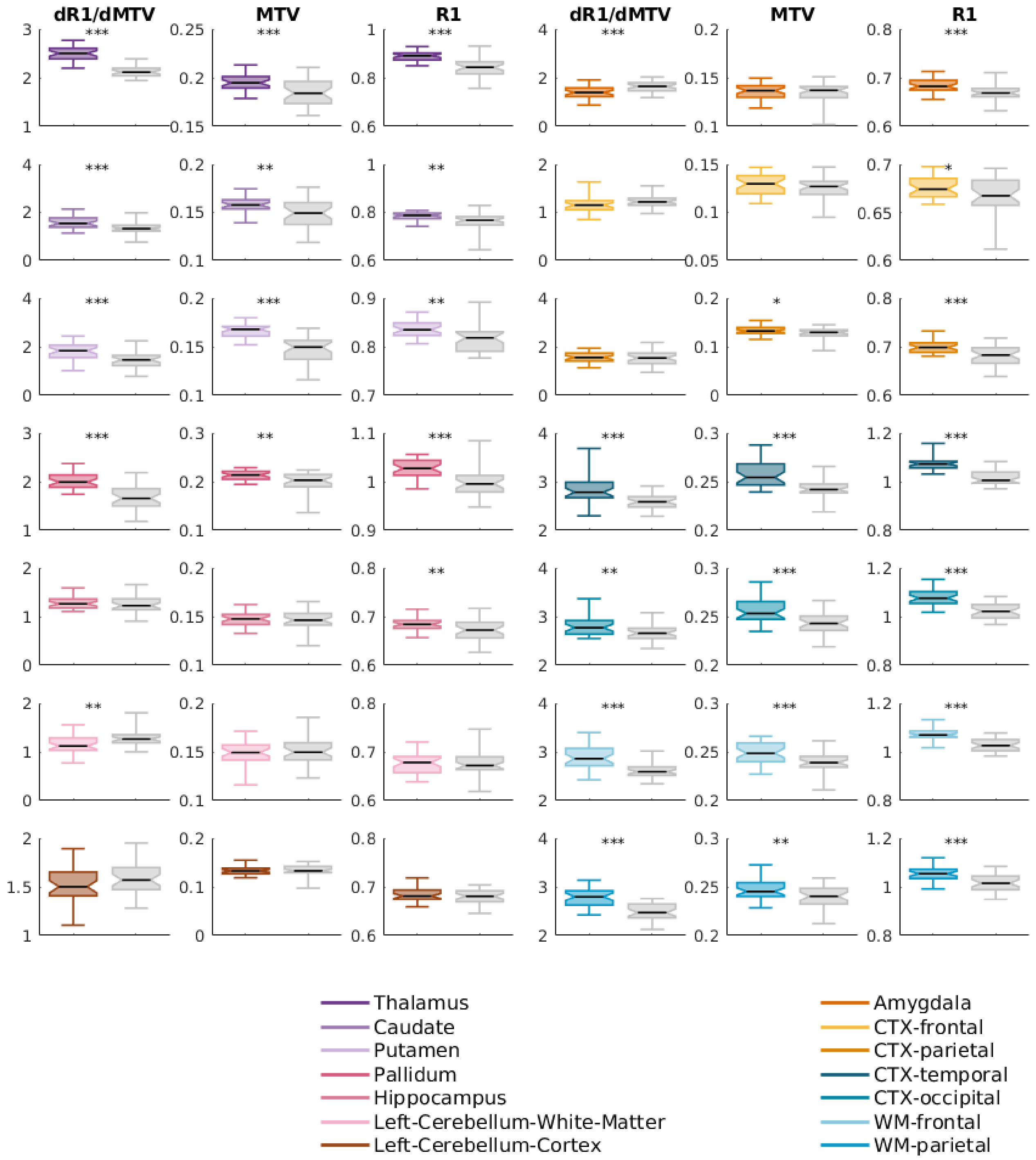

Supplementary Figure 23: Aging-related changes in R1 revealed by the MDM approach – R2 correction.*
Comparison of MRI-driven measurements of 18 older adults (marked in gray) and 17 younger adults (marked with different colors) in different brain regions (see legend) when MTV was corrected for R2 (MTV_C).*
Aging-related changes revealed by R1 measurement are presented in the R1 columns. The separation to chemophysical and water related constituents estimated by the MTV_C derivative and MTV_C respectively is shown in the $dR1/dMTV_C$ and MTV_C columns. The statistical significance of the differences between the groups was estimated using a two-sample t-test and was corrected for multiple comparisons using the FDR method.
** P < 0.05;*
*** P < 0.01;*
**** P < 0.001.*

Supplementary Figure 24- Aging-related changes in MTsat revealed by the MDM approach – R2* correction.

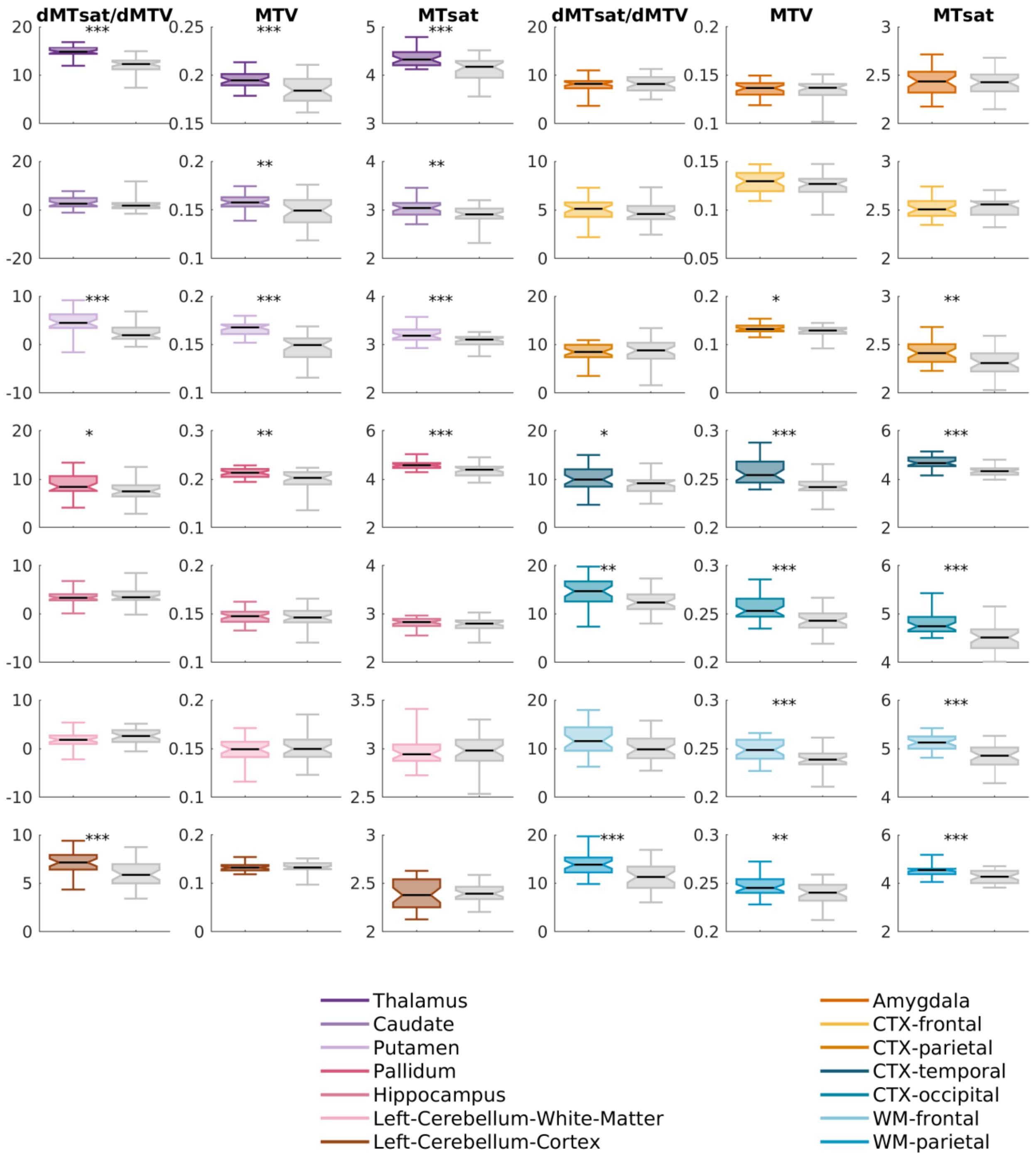

Supplementary Figure 24: Aging-related changes in MTsat revealed by the MDM approach – R2 correction.*
Comparison of MRI-driven measurements of 18 older adults (marked in gray) and 17 younger adults (marked with different colors) in different brain regions (see legend) when MTV was corrected for R2 (MTV_C).*
Aging-related changes revealed by MTsat measurement are presented in the MTsat columns. The separation to chemophysical and water related constituents estimated by the MTV_C derivative and MTV_C respectively is shown in the dMTsat/$dMTV_C$ and MTV_C columns. The statistical significance of the differences between the groups was estimated using a two-sample t-test and was corrected for multiple comparisons using the FDR method.
** $P < 0.05$;*
*** $P < 0.01$;*
**** $P < 0.001$.*

Supplementary Figure 25- Aging-related changes in MD revealed by the MDM approach – R2* correction.

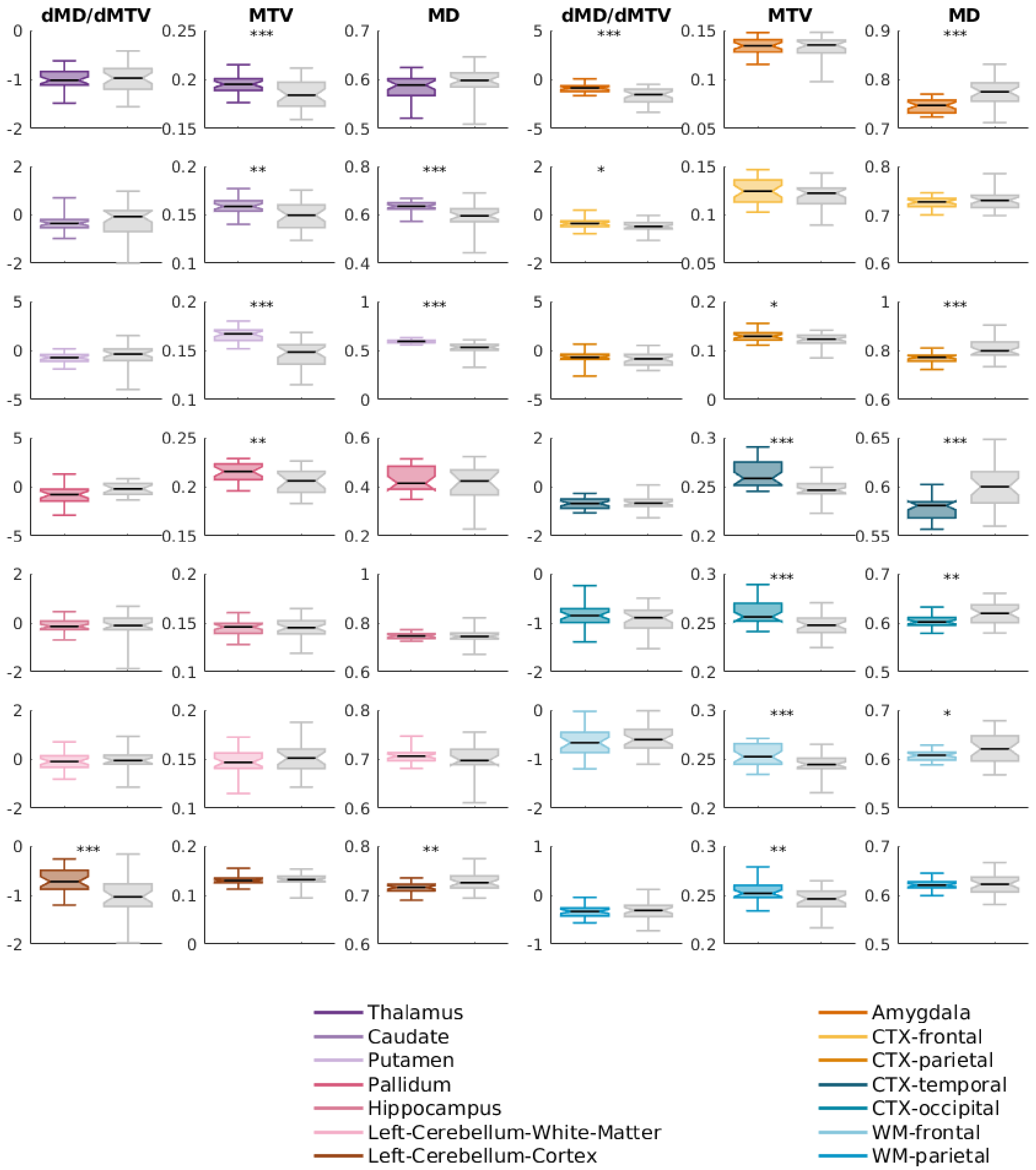

Supplementary Figure 25: Aging-related changes in MD revealed by the MDM approach – R2 correction.* *Comparison of MRI-driven measurements of 17 older adults (marked in gray) and 17 younger adults (marked with different colors) in different brain regions (see legend) for the case in which MTV was corrected for R2* (MTV_C). Aging-related changes revealed by MD measurement are presented in the MD columns. The separation to chemophysical and water related constituents estimated by the MTV_C derivative and MTV_C respectively is shown in the dMD/dMTV_C and MTV_C columns. The statistical significance of the differences between the groups was estimated using a two-sample t-test and was corrected for multiple comparisons using the FDR method.*
** P < 0.05;*
*** P < 0.01;*
**** P < 0.001*

Supplementary Figure 26- Aging-related changes in R2 revealed by the MDM approach – R2* correction.

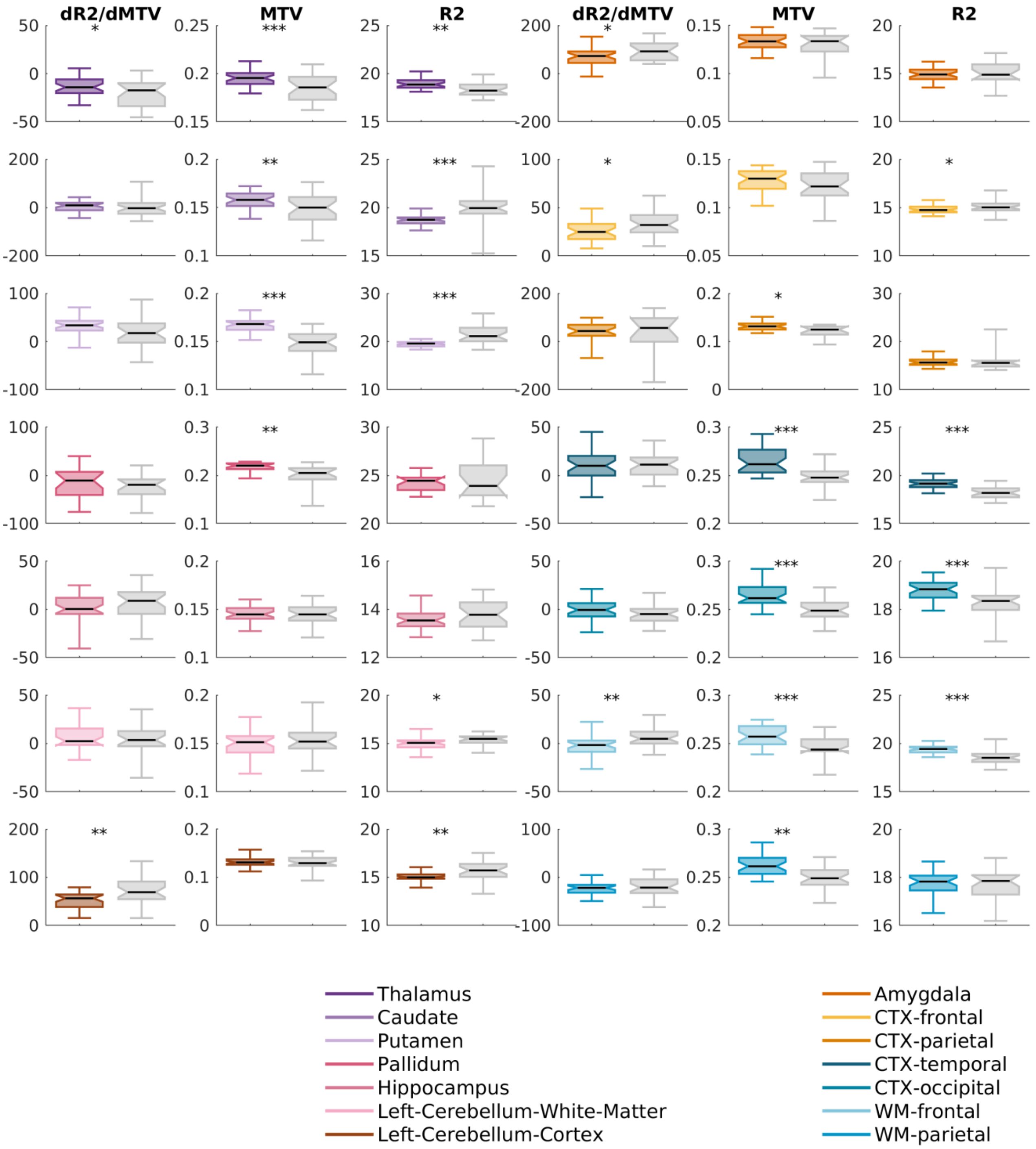

Supplementary Figure 26: Aging-related changes in R2 revealed by the MDM approach – R2 correction.*
Comparison of MRI-driven measurements of 15 older adults (marked in gray) and 17 younger adults (marked with different colors) in different brain regions (see legend) for the case in which MTV was corrected for R2 (MTV_C). Aging-related changes revealed by R2 measurement are presented in the R2 columns. The separation to chemophysical and water related constituents estimated by the MTV_C derivative and MTV_C respectively is shown in the dR2/dMTV_C and MTV_C columns. The statistical significance of the differences between the groups was estimated using a two-sample t-test and was corrected for multiple comparisons using the FDR method.*
** $P < 0.05$;*
*** $P < 0.01$;*
**** $P < 0.001$.*

Supplementary Figure 27- The biological interpretation of the MDM approach – following R2* correction.

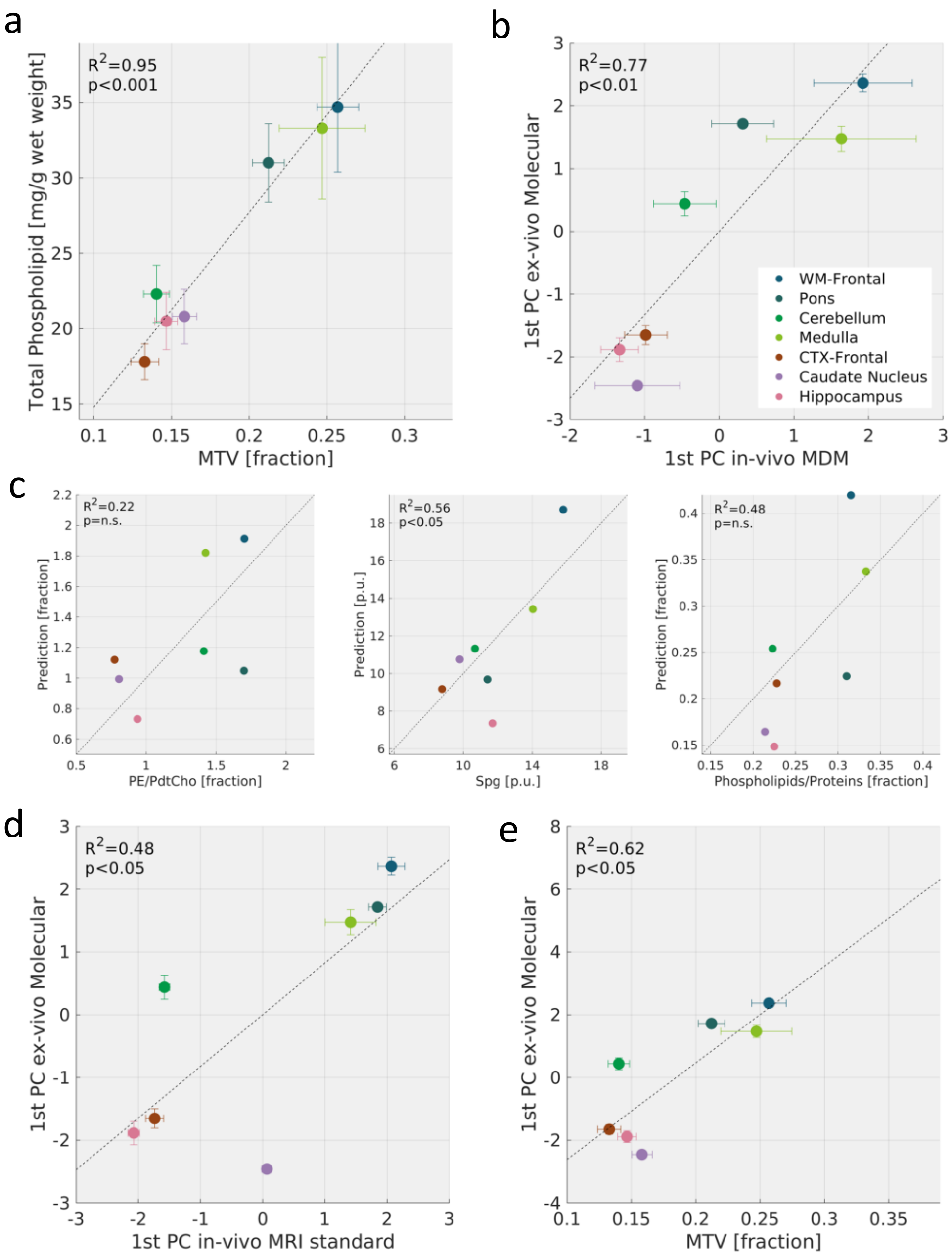

Supplementary Figure 27: The biological interpretation of the MDM approach – following R2* the correction. The agreement between MDM and histology presented in figure 3 still holds after MTV was corrected for R2*.

__a)__ Comparison of the total phospholipid content derived from the literature (y-axis) to the average MTV measurement across 15 young subjects (x-axis) in 7 different brain regions (colored data points) after correction for R2.*

__b)__ The projection of different brain regions (colored data points) on the 1st principle component (PC) of molecular variability (y-axis, derived from the literature) vs. their projection on the 1st principle component (PC) of MDM (x-axis, averaged over 15 young subjects). The data corrected for R2 was projected on the same direction as in figure 3b.*

__c)__ MDM-based prediction of different molecular features (y-axes, derived from the literature) compared to their true value (x-axes) in different brain regions (colored data points) after correction for R2. MDM-based predictions were computed through a weighted linear sum of the MTV derivatives of R1 and MTsat (as PCA indicates they account for most of the variability in MDM across the brain). This linear model was fitted using leave-one-out cross validation.*

__d)__ The correlation of the molecular variability with standard qMRI parameters after correction for R2. The projection of different brain regions on the 1st PC of molecular variability (y-axis, derived from the literature) vs. their projection on the 1st PC of standard qMRI parameters (x-axis, averaged over 15 young subjects).*

__e)__ The correlation of the molecular variability with after correcting for R2. The projection of different brain regions (colored data points) on the 1st principal component (PC) of molecular variability (y-axis, derived from the literature) vs. their MTV values (x-axis, averaged over 15 young subjects). Adjusted R^2 values presented for the entire figure.*

Supplementary Figure 28- Examination of the linear relationship of qMRI parameters and 1/PD.

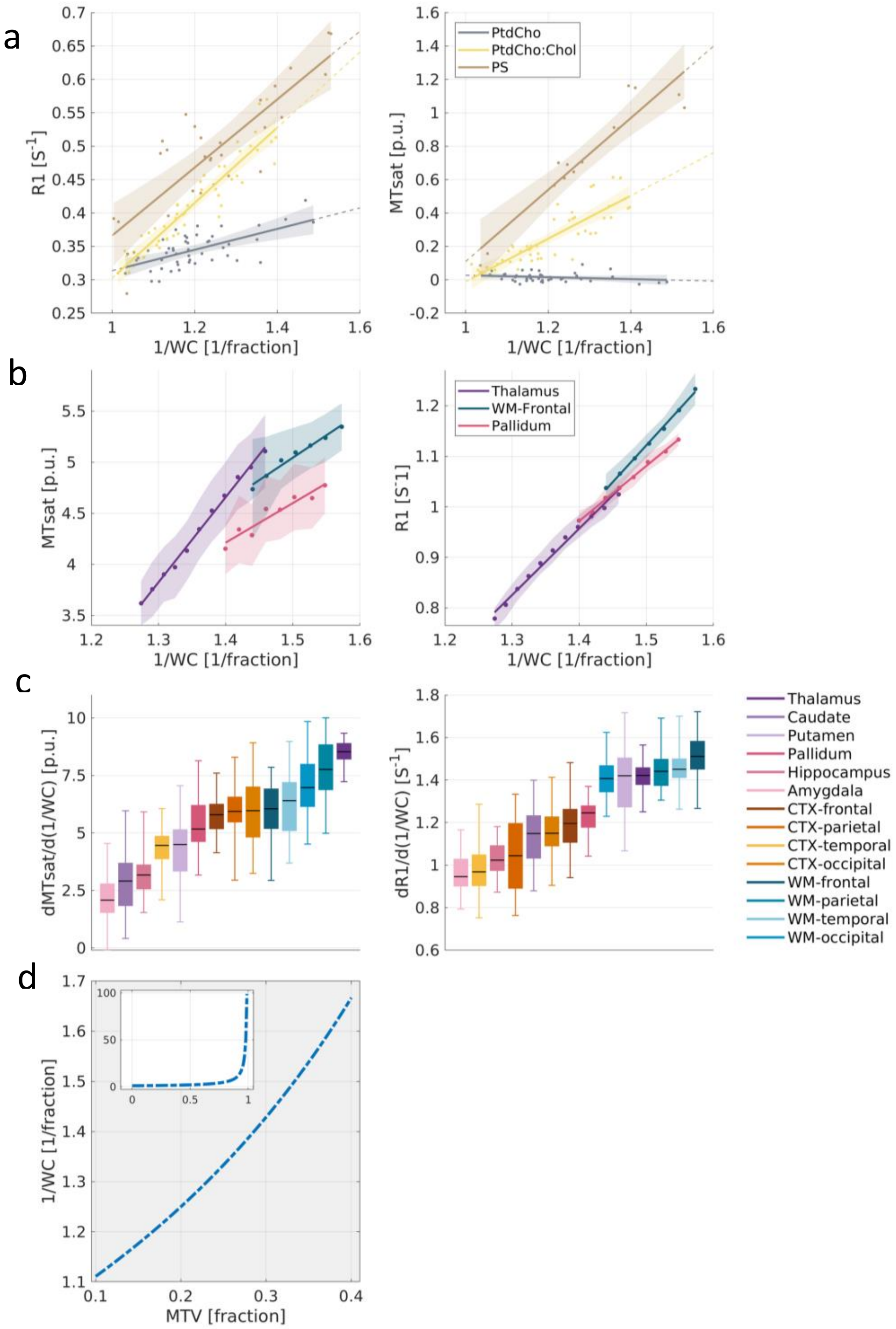

Supplementary Figure 28: Examination of the linear relationships of qMRI parameters and 1/Water Content (1/WC).
a) qMRI parameters are linear with 1/WC in lipid phantoms. *The dependency of MTsat and R1 on 1/WC for three lipids. Colored dots represent the median of different lipid samples with varying water concentrations. Shaded areas represent the 95% confidence bounds for the fitted slopes.*
b) qMRI parameters are linear with 1/WC in the human brain. *The dependency of MTsat and R1 on 1/WC in three brain regions of a single subject. For each region, 1/WC values were pooled into bins (dots are the median of each bin; shaded area is the median absolute deviation), and a linear fit was calculated (colored lines). The slopes of the linear fit represent the 1/WC derivatives of R1 and MTsat and vary across brain regions.*
c) Similarly to MTV, the 1/WC derivatives of qMRI parameters are region-specific. *The figure shows the reliability of the 1/WC derivatives of MTsat (C) and R1 (D) across young subjects (23). Different colors represent 14 brain regions (see legend). Edges of each box represent the 25th, and 75th percentiles, median is in black, and whiskers extends to extreme data points. Different brain regions show distinct MTV derivatives.*
d) In a biological range, 1/WC varies almost linearly with MTV. *Comparison of MTV and 1/WC in a biologically-relevant MTV range (1/WC=1/(1-MTV)). The inset shows this relationship for the entire range of possible MTV values.*

Supplementary Figure 29- The "mosaic" nature of aging trajectories- additional MDM dimensions.

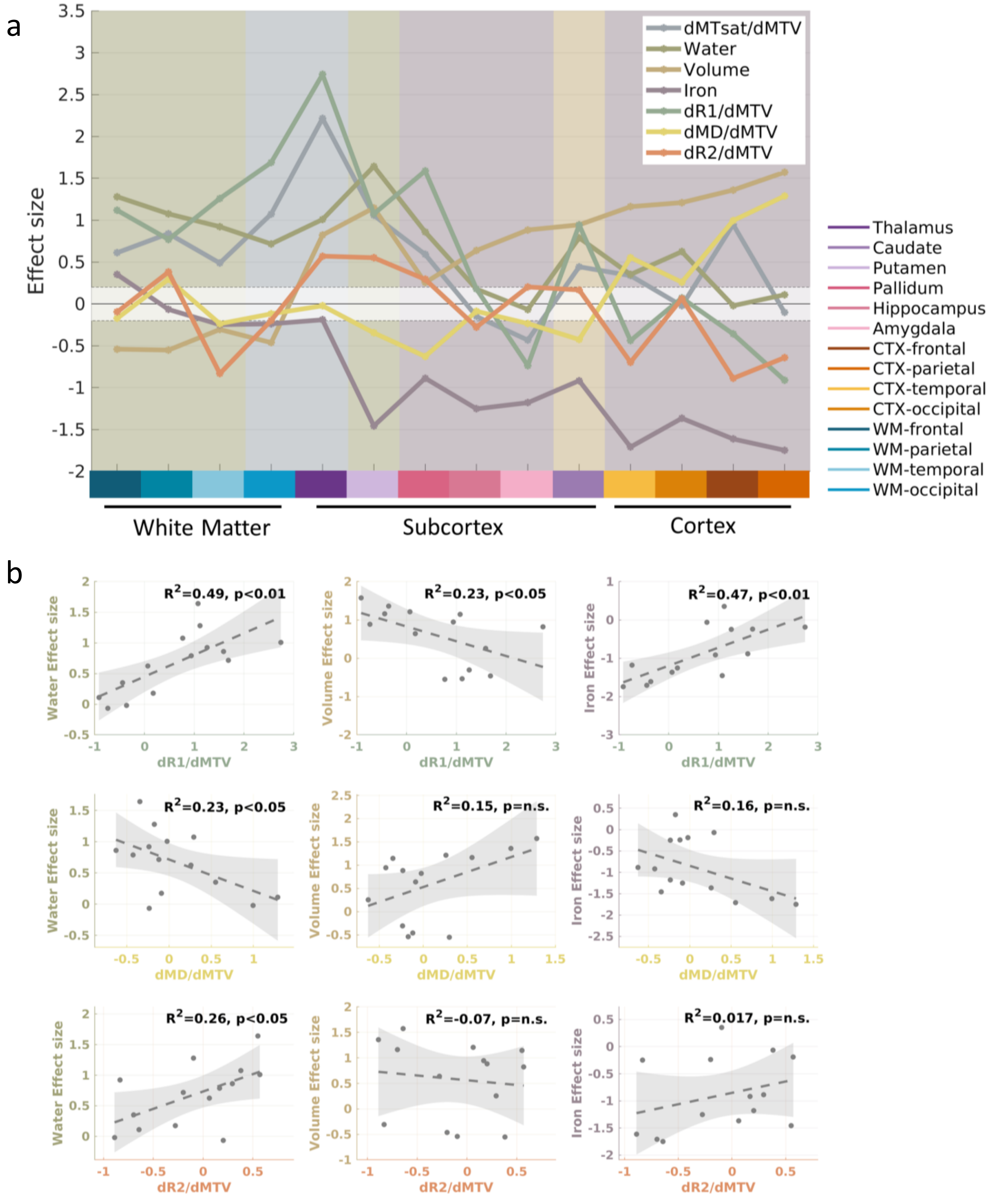

Supplementary Figure 29: The "mosaic" nature of aging trajectories- additional MDM dimensions.
a) Distinct spatial patterns of different aging markers throughout the brain. *Different colors represent seven markers of brain aging. The MTV derivatives of four qMRI parameters are presented (instead of only dMTsat/dMTV in Figure 8). The water and iron markers were estimated using MTV and R2* respectively. The x-axis represents different brain regions (see legend). The y-axis shows the effect size of age-related changes between the younger and older groups evaluated using Cohen's d. Shaded background colors are as in figure 8a. Note that except from the thalamus, the additional MDM dimensions did not change the trend showed in the background of the largest effect size for each brain region. Dashed line marks the limit of small effect size (d=0.2). MTV derivatives and MTV were corrected for R2* effects.*
b) The spatial correlation between aging-related changes in volume, iron and water to all MDM dimensions. *Each plot shows the correlation in the effect sizes of two aging markers across different brain regions. Shaded areas represent the 95% confidence bounds for the fitted linear model.* Adjusted R^2 values presented.

Supplementary Figure 30- Distinct molecular aging trajectories- comparison of different MDM dimensions.

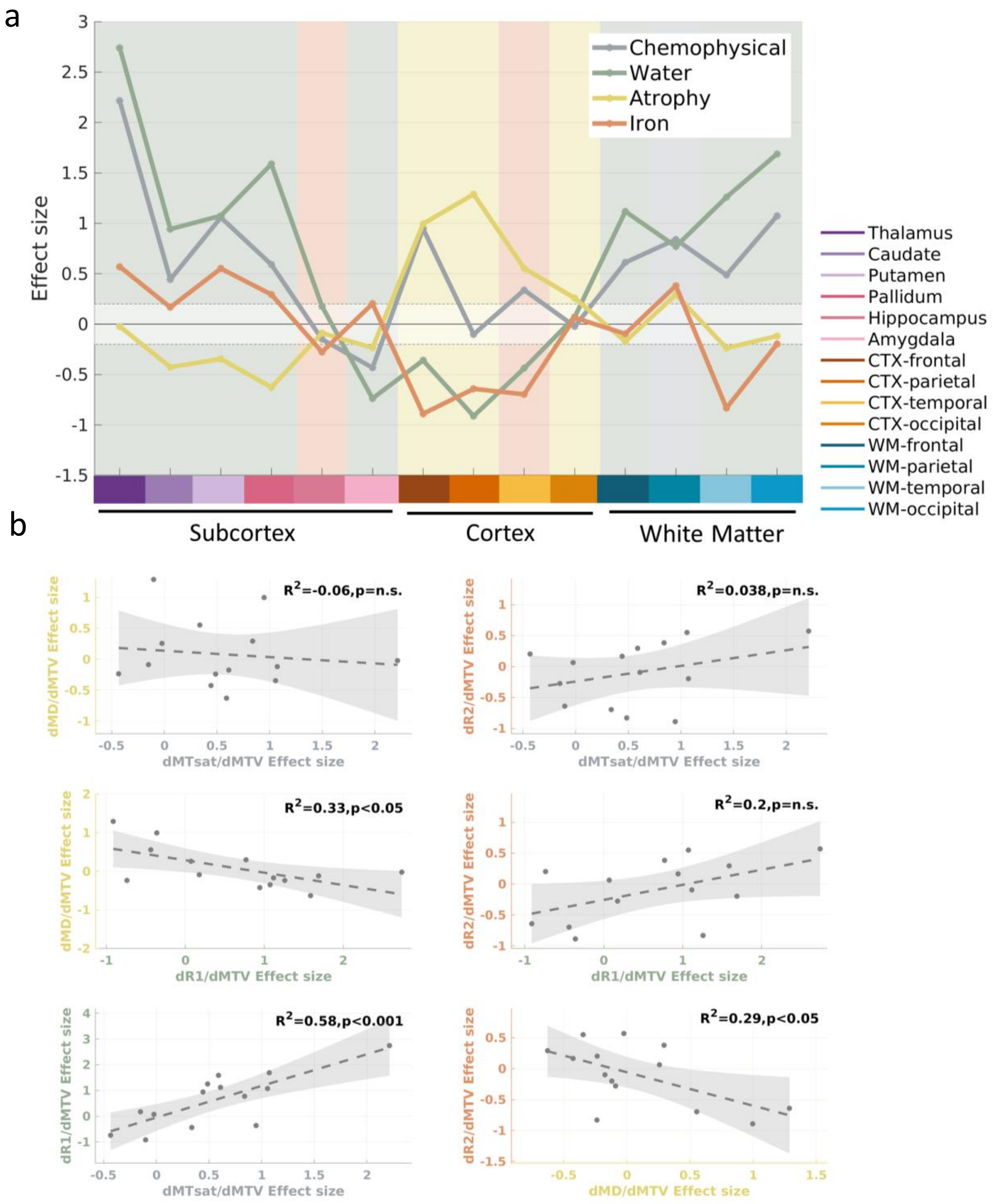

Supplementary Figure 30: Distinct molecular aging trajectories- comparison of different MDM dimensions.

a) Distinct spatial patterns of different molecular aging markers throughout the brain. *Different colors represent four MTV derivatives (MDM dimensions). The x-axis represents different brain regions (see legend). The y-axis shows the effect size of age-related changes between the younger and older groups evaluated using Cohen's d. Shaded background colors indicate which of the four markers has the largest effect size for each brain region. Dashed line marks the limit of small effect size (d=0.2). All MTV derivatives were corrected for R2* effects.*

b) The spatial correlation between aging-related changes in different MTV derivatives. *Each plot shows the correlation in the effect sizes of two MTV derivatives across different brain regions. Shaded areas represent the 95% confidence bounds for the fitted linear model. Adjusted R^2 values presented.*

References

1. Söderberg, M., Edlund, C., Kristensson, K. & Dallner, G. Lipid Compositions of Different Regions of the Human Brain During Aging. *J. Neurochem.* **54,** 415–423 (1990).
2. Ben-David, E. & Shifman, S. Networks of Neuronal Genes Affected by Common and Rare Variants in Autism Spectrum Disorders. *PLoS Genet.* **8,** e1002556 (2012).
3. Solodkin, A., Veldhuizen, S. D. & Van Hoesen, G. W. Contingent vulnerability of entorhinal parvalbumin-containing neurons in Alzheimer's disease. *J. Neurosci.* **16,** 3311–21 (1996).
4. Wei, F. *et al.* Ion Channel Genes and Epilepsy: Functional Alteration, Pathogenic Potential, and Mechanism of Epilepsy. *Neurosci. Bull.* **33,** 455–477 (2017).
5. Liu, W., Geng, L. & Chen, Y. MiR-19b alleviates MPP+-induced neuronal cytotoxicity via targeting the HAPLN4/MAPK pathway in SH-SY5Y cells. *RSC Adv.* **8,** 10706–10714 (2018).
6. Callaghan, M. F., Helms, G., Lutti, A., Mohammadi, S. & Weiskopf, N. A general linear relaxometry model of R1 using imaging data. *Magn. Reson. Med.* **73,** 1309–14 (2015).
7. Langkammer, C. *et al.* Quantitative MR Imaging of Brain Iron: A Postmortem Validation Study. *Radiology* **257,** 455–462 (2010).
8. Zecca, L., Youdim, M. B. H., Riederer, P., Connor, J. R. & Crichton, R. R. Iron, brain ageing and neurodegenerative disorders. *Nat. Rev. Neurosci.* **5,** 863–873 (2004).
