# Supplementary material for: Disentangling molecular alterations from water-content changes in the aging human brain using quantitative MRI
Source: Nat Commun. 2019 Jul 30;10:3403. doi: 10.1038/s41467-019-11319-1 (PMC6667463; doi:10.1038/s41467-019-11319-1)
Supplement: Supplementary file 3 — Reporting Summary [file 41467_2019_11319_MOESM3_ESM.pdf]

nature research

Corresponding author(s): Aviv Mezer

Last updated by author(s): Jun 21, 2019

Reporting Summary

Nature Research wishes to improve the reproducibility of the work that we publish. This form provides structure for consistency and transparency in reporting. For further information on Nature Research policies, see Authors & Referees and the Editorial Policy Checklist.

Statistics

For all statistical analyses, confirm that the following items are present in the figure legend, table legend, main text, or Methods section.

n/a	Confirmed	
☐	☒	The exact sample size (*n*) for each experimental group/condition, given as a discrete number and unit of measurement
☐	☒	A statement on whether measurements were taken from distinct samples or whether the same sample was measured repeatedly
☐	☒	The statistical test(s) used AND whether they are one- or two-sided<br>*Only common tests should be described solely by name; describe more complex techniques in the Methods section.*
☐	☒	A description of all covariates tested
☐	☒	A description of any assumptions or corrections, such as tests of normality and adjustment for multiple comparisons
☐	☒	A full description of the statistical parameters including central tendency (e.g. means) or other basic estimates (e.g. regression coefficient) AND variation (e.g. standard deviation) or associated estimates of uncertainty (e.g. confidence intervals)
☐	☒	For null hypothesis testing, the test statistic (e.g. *F*, *t*, *r*) with confidence intervals, effect sizes, degrees of freedom and *P* value noted<br>*Give P values as exact values whenever suitable.*
☐	☒	For Bayesian analysis, information on the choice of priors and Markov chain Monte Carlo settings
☐	☒	For hierarchical and complex designs, identification of the appropriate level for tests and full reporting of outcomes
☐	☒	Estimates of effect sizes (e.g. Cohen's *d*, Pearson's *r*), indicating how they were calculated

Our web collection on statistics for biologists contains articles on many of the points above.

Software and code

Policy information about availability of computer code

Data collection	Data was collected on a 3T Siemens MAGNETOM Skyra scanner equipped with a 32-channel head receive-only coil at the ELSC neuroimaging unit at the Hebrew University.<br>LImidomics were collected using Thin Layer Chromatography (TLC). This analysis was conducted in the Bert Strassburger Lipid Center, Sheba, Tel Hashomer.
Data analysis	The software described in the paper for calculating MDM was developed on Matlab 2017b.<br>qMRI maps were generated using the following softwares:<br>1) mrQ (https://github.com/mezera/mrQ)<br>2) vistasoft (https://github.com/vistalab/vistasoft/wiki)<br>3) MPM (https://github.molgen.mpg.de/hMRI-group/Toolbox)<br>4) Freesurfer (https://surfer.nmr.mgh.harvard.edu/)<br>5) FSL (https://fsl.fmrib.ox.ac.uk/fsl/fslwiki)<br>6) ANTs (http://stnava.github.io/ANTs/)<br>7) EMC (https://cai2r.net/resources/software/emc-based-t2-mapping-package)<br><br>Lipidomics analysis was quantified using Optiquant (Epson V700)<br><br>* A toolbox for computing the MDM signatures is freely available at GitHub. The code for generating the figures of the paper, including the relevant data, is also available as a GitHub repository.

For manuscripts utilizing custom algorithms or software that are central to the research but not yet described in published literature, software must be made available to editors/reviewers. We strongly encourage code deposition in a community repository (e.g. GitHub). See the Nature Research guidelines for submitting code & software for further information.

Data

Policy information about availability of data

All manuscripts must include a data availability statement. This statement should provide the following information, where applicable:

- Accession codes, unique identifiers, or web links for publicly available datasets
- A list of figures that have associated raw data
- A description of any restrictions on data availability

A toolbox for computing the MDM signatures is freely available at GitHub. The code for generating the figures of the paper, including the relevant data, is also available as a GitHub repository. The datasets generated during and/or analyzed during the current study are available from the corresponding author on reasonable request.

Field-specific reporting

Please select the one below that is the best fit for your research. If you are not sure, read the appropriate sections before making your selection.

☒ Life sciences ☐ Behavioural & social sciences ☐ Ecological, evolutionary & environmental sciences

For a reference copy of the document with all sections, see nature.com/documents/nr-reporting-summary-flat.pdf

Life sciences study design

All studies must disclose on these points even when the disclosure is negative.

Sample size	Human measurements were performed on 23 young adults (aged 27 ± 2 years, 11 females), and 18 older adults (aged 67 ± 6 years, 5 females).
Data exclusions	For five subjects (4 young, 1 old) we failed to acquire non-diffusion-weighted images with reversed phase-encode blips and they were excluded from the diffusion analysis (see methods).
Replication	We preformed scan re-scan tests for the human subjects and the phantom system. qMRI parameters were found to be highly reproducible.
Randomization	Participants were allocated to groups according to their age.
Blinding	During data collection the investigator met the participants and hence was aware of their age.

Reporting for specific materials, systems and methods

We require information from authors about some types of materials, experimental systems and methods used in many studies. Here, indicate whether each material, system or method listed is relevant to your study. If you are not sure if a list item applies to your research, read the appropriate section before selecting a response.

Materials & experimental systems

n/a	Involved in the study	
☒	☐	Antibodies
☒	☐	Eukaryotic cell lines
☒	☐	Palaeontology
☒	☐	Animals and other organisms
☐	☒	Human research participants
☒	☐	Clinical data

Methods

n/a	Involved in the study	
☒	☐	ChIP-seq
☒	☐	Flow cytometry
☐	☒	MRI-based neuroimaging

Human research participants

Policy information about studies involving human research participants

Population characteristics	Human measurements were performed on 23 young adults (aged 27 ± 2 years, 11 females), and 18 older adults (aged 67 ± 6 years, 5 females).
Recruitment	Participants were recruited using through printed and electronic advertisements. The Helsinki Ethics Committee of Hadassah Hospital, Jerusalem, Israel approved the experimental procedure. Written informed consent was obtained from each participant prior to the procedure.
Ethics oversight	The Helsinki Ethics Committee of Hadassah Hospital, Jerusalem, Israel approved the experimental procedure.

Note that full information on the approval of the study protocol must also be provided in the manuscript.

Magnetic resonance imaging

Experimental design

Design type	As we use quantitative MRI this is not relevant.
Design specifications	As we use quantitative MRI this is not relevant.
Behavioral performance measures	As we use quantitative MRI this is not relevant.

Acquisition

Imaging type(s)	structural, diffusion
Field strength	3 Tesla
Sequence & imaging parameters	MRI Acquisition:<br>Quantitative R1, R2* & MTV mapping: 3D Spoiled gradient (SPGR) echo images were acquired with different flip angles (α = 4°, 10°, 20° and 30°). Each image included 5 equally spaced echoes (TE=3.34-14.02 ms) and the TR was 19 ms (except for 6 young subjects for which the scan included only one TE=3.34 ms). The scan resolution was 1 mm isotropic. For calibration, we acquired additional spin-echo inversion recovery scan with an echo-planar imaging (EPI) read-out (SEIR-epi). This scan was done with a slab-inversion pulse and spatial-spectral fat suppression. For SEIR-epi, the TE/TR was 49/2920 ms. TI were 200, 400, 1,200, and 2,400 ms. We used 2-mm in-plane resolution with a slice thickness of 3 mm. The EPI readout was performed using 2× acceleration.<br>Quantitative T2: Multi-SE images were acquired, with 10 equally spaced spin echoes between 12 ms to 120 ms. The TR was 4.21 s. The scan resolution was 2 mm isotropic. T2 scans of four subjects (1 young, 3 old) were excluded from the analysis due to motion.<br>Quantitative MTsat: 3D Spoiled gradient (SPGR) echo image with an additional MT pulse. The flip angle was 10°, the TE/TR was 3.34/2700 ms. The scan resolution was 1 mm isotropic.<br>Anatomical images: 3D magnetization prepared rapid gradient echo (MP-RAGE) scans were acquired for 24 of the subjects (14 from the younger subjects, 10 from the older subjects). The scan resolution was 1 mm isotropic, the TE/TR was 2.98/2300 ms. Magnetization Prepared 2 Rapid Acquisition Gradient Echoes (MP2RAGE) scans were acquired for the rest of the subjects. The scan resolution was 1 mm isotropic, the TE/TR was 2.98/5000 ms.
Area of acquisition	Whole brain

Diffusion MRI ☒ Used ☐ Not used

Parameters	Diffusion mapping: Whole-brain DTI measurements were performed using a diffusion-weighted spin-echo EPI sequence with isotropic 1.5-mm resolution. Diffusion weighting gradients were applied at 64 directions and the strength of the diffusion weighting was set to b=2000s/mm2 (TE/TR=95.80/6,000ms, G=45mT/m, δ=32.25ms, Δ=52.02ms). The data includes eight non-diffusion-weighted images (b=0). In addition, we collected non-diffusion-weighted images with reversed phase-encode blips. For five subjects (4 young, 1 old) we failed to acquire this correction data and they were excluded from the diffusion analysis.

Preprocessing

Preprocessing software	qMRI maps were generated using the following softwares:<br>1) mrQ (https://github.com/mezera/mrQ)<br>2) vistasoft (https://github.com/vistalab/vistasoft/wiki)<br>3) MPM (https://github.molgen.mpg.de/hMRI-group/Toolbox)<br>4) Freesurfer (https://surfer.nmr.mgh.harvard.edu/)<br>5) FSL (https://fsl.fmrib.ox.ac.uk/fsl/fslwiki)<br>6) ANTs (http://stnava.github.io/ANTs/)<br>7) EMC (https://cai2r.net/resources/software/emc-based-t2-mapping-package)
Normalization	As we use quantitative MRI this is not relevant.
Normalization template	As we did all our analyses in subject's space this is not relevant.
Noise and artifact removal	Coils sensitivity and B1 inhomogeneities were removed using the mrQ software (https://github.com/mezera/mrQ)
Volume censoring	As we use quantitative MRI this is not relevant.

Statistical modeling & inference

Model type and settings	As we use quantitative MRI this is not relevant.
Effect(s) tested	As we use quantitative MRI this is not relevant.

Specify type of analysis: ☒ Whole brain ☐ ROI-based ☐ Both

Statistic type for inference
(See Eklund et al. 2016)

As we use quantitative MRI this is not relevant.

Correction

The statistical significance of the differences between the age groups was computed using an independent-sample t-test (alpha = 0.05, both right and left tail) and was corrected for multiple comparisons using the false discovery rate (FDR) method.
R^2 measurements were adjusted for the number of data points.

Models & analysis

n/a	Involved in the study
☒	☐ Functional and/or effective connectivity
☒	☐ Graph analysis
☐	☒ Multivariate modeling or predictive analysis

Multivariate modeling and predictive analysis

We used the MDM approach to predict the molecular composition of the human brain (Fig 3C). For this, we used Supplementary Equation 5 and a cross-validation process; prediction for each brain area was computed by removing it from the system and solving for the other brain areas.
The calculation involved 7 human brain molecular features (%PE, %PS, %PtdCho %PI, %Spg, phospholipids/proteins, phospholipids/cholesterol), and 4 MDM measurements (dR1/dMTV, dMTsat/dMTV, dR2dMTV, dMD/dMTV). PCA was used to reduce the dimensionality of the system and avoid over-fitting. We identified the 3 molecular features with the largest loadings on the first PC of molecular variability. The fractions of the lipids PE and PtdCho had the largest loadings, and we used the ratio between them as it was found to better characterize individual brain regions. The two other features with large loadings were the fraction of the lipid Spg and the phospholipids/proteins ratio. We predicted these 3 human brain molecular features using the MTV derivatives that account for most of the MDM variability. The two measurements with the largest loadings on the first PC of MDM were dR1/dMTV and dMTsat/dMTV.
